# Synergistic inhibition of metastatic breast cancer by dual-chemotherapy with excipient-free rhein/DOX nanodispersions

**DOI:** 10.1186/s12951-020-00679-2

**Published:** 2020-08-26

**Authors:** Ruoning Wang, Yujie Yang, Mengmeng Yang, Dandan Yuan, Jinyu Huang, Rui Chen, Honglan Wang, Lihong Hu, Liuqing Di, Junsong Li

**Affiliations:** 1 School of Pharmacy, Nanjing University of Chinese Medicine, 138 Xianlin Avenue, Nanjing, 210023 China; 2 Jiangsu Engineering Research Center for Efficient Delivery System of TCM, Nanjing, China; 3 Jiangsu Key Laboratory for Functional Substance of Chinese Medicine, Nanjing, China

**Keywords:** Metastatic cancer, Synergistic antiproliferative effect, Rhein (RHE) and doxorubicin (DOX), Excipient-free nanodispersions

## Abstract

**Background:**

The management of metastatic cancer remains a major challenge in cancer therapy worldwide. The targeted delivery of chemotherapeutic drugs through rationally designed formulations is one potential therapeutic option. Notably, excipient-free nanodispersions that are entirely composed of pharmaceutically active molecules have been evaluated as promising candidates for the next generation of drug formulations. Formulated from the self-assembly of drug molecules, these nanodispersions enable the safe and effective delivery of therapeutic drugs to local disease lesions. Here, we developed a novel and green approach for preparing nanoparticles via the self-assembly of rhein (RHE) and doxorubicin (DOX) molecules, named RHE/DOX nanoparticles (RD NPs); this assembly was associated with the interaction force and did not involve any organic solvents.

**Results:**

According to molecular dynamics (MD) simulations, DOX molecules tend to assemble around RHE molecules through intermolecular forces. This intermolecular retention of DOX was further improved by the nanosizing effect of RD NPs. Compared to free DOX, RD NPs exerted a slightly stronger inhibitory effect on 4T1 cells in the scratch healing assay. As a dual drug-loaded nanoformulation, the efficacy of RD NPs against tumor cells in vitro was synergistically enhanced. Compared to free DOX, the combination of DOX and RHE in nanoparticles exerted a synergistic effect with a combination index (CI) value of 0.51 and showed a stronger ability to induce cell apoptosis. Furthermore, the RD NP treatment not only effectively suppressed primary tumor growth but also significantly inhibited tumor metastasis both in vitro and in vivo, with a better safety profile.

**Conclusions:**

The generation of pure nanodrugs via a self-assembly approach might hold promise for the development of more efficient and novel excipient-free nanodispersions, particularly for two small molecular antitumor drugs that potentially exert synergistic antiproliferative effects on metastatic breast cancer.

## Background

The American Cancer Society and the National Cancer Institute have reported that more than 3.8 million women in the United States are diagnosed with invasive breast cancer [1]. Approximately 25% of these patients succumb to their disease due to the progression and development of distant metastases in the lungs and other tissues. During the process of metastasis, tumor cells move systemically and spread into other organs; this process results in a poor prognosis and increases the patient’s mortality [2, 3]. In recent years, the new and efficient nanodrug delivery system has become an emerging method for treating cancer [4]. The unique advantages of nanodrug delivery systems, including targeted drug delivery [5–8] and sustained-release drug delivery, have improved the solubility of insoluble drugs and reduced adverse drug reactions, potentially allowing them to target the tumor tissue in a specific and controlled manner.

Furthermore, patients with hormone receptor-negative metastatic breast cancer are treated with systemic chemotherapy [9]. However, single-agent chemotherapy tends to fail, ultimately leading to the death of patients [10]. Due to the limitations of single-agent chemotherapy, the rational combination of two or more drugs targeted at different cellular pathways has attracted attention to improve anticancer effects during the treatment of metastatic cancer [11]. Over the past few decades, combination chemotherapy has shown superior efficacy to single-agent chemotherapy. Nevertheless, due to the distinct pharmacokinetics of each agent, the effective drug dosage and ratio of the conventional ‘cocktail’ chemotherapy are difficult to control [12]. Moreover, unexpected side effects of ‘cocktail’ chemotherapy have been reported in clinical trials [13]. Thus, the efficient codelivery of multiple therapeutic agents to a target tissue with a controlled dose ratio and synergistic efficacy is highly desirable for future clinical translation. Many nanosized codelivery architectures, such as liposomes [14], micelles [15, 16], mesoporous silica nanoparticles [17] and hydrogels [18], have been reported to address this need. For instance, the all-in-one brush-arm star polymer nanoparticles (NPs) were designed by ring-opening metathesis polymerization, which generated precise molar ratios of doxorubicin (DOX) [19], camptothecin [20] and cisplatin [21]. Similarly, liposomes coloaded with oxaliplatin and irinotecan were developed that achieved synchronized delivery and exhibited superior antitumor activity compared to the free drugs [22].

Although a large number of nanomedicine drug delivery systems have been reported to date, only a few have been used in the clinic. One major obstacle for clinical usage is that most of these methods require a large quantity of excipient materials and involve complicated processes [23]. The usage of excipient material usually leads to extensive clinical trials and extra FDA approvals. Moreover, the production of nanomedicines is often too complicated to develop in a scaled up manufacturing setting [24]. Therefore, a “green” approach to designing nanomedicines without “toxic” excipients must be developed. The nano-assembly strategy for designing excipient-free nanodispersions may be applied to nanoparticulate anticancer agents to increase the therapeutic effects, which is the purpose of green pharmaceuticals [25, 26]. Formulations and processes have been developed to significantly eliminate the use and generation of hazardous substances [27]. Recently, a carrier-free dual drug delivery system generated by the self-nanocrystallization of drugs was developed by Kushwah et al. [2]. Using DOX as a stabilizing agent, spherically assembled particles with a uniform size were prepared to increase the water solubility of 10-hydroxycamptothecin (HCPT). HCPT combined with the photosensitizer chlorin e6 was employed as a stabilizer to obtain stable rod-like nanoparticles using a nanoprecipitation method. However, these studies have mainly focused on improving the solubility of drugs with poor solubility and have ignored their compatibility. Therefore, excipient-free “pure” nanodrugs might become the next-generation nanomedicine [28].

DOX is widely used to treat solid tumors, such as breast, lung and ovarian cancer; this compound enters cells by diffusing across the plasma membrane, entering the nucleus and causing DNA damage [25]. DOX further suppresses the activity of P53, Bcl-2, Bax and Caspase 3, leading to the apoptosis of cancer cell [29]. Nevertheless, DOX also causes toxicity in the liver, kidney and heart. Meanwhile, the toxic effects of DOX on the cardiac muscles are cumulative and irreversible, which limit the use of DOX [30]. Rhein (RHE), a bioactive molecule derived from the herb rhubarb, exhibits potent anti-inflammatory and antioxidant activities and is safe, even at high doses. The anticancer mechanisms of RHE include blocking the transcription factor NF-κB and targeting the MAPK and p-AKT pathways. However, because of the hydrophobic property of RHE, vascular administration would be extremely difficult [15]. These disadvantages hamper the ability of researchers to design optimal medical regimes for the treatment of diseases and call for the development of a nano-assembly strategy.

**Fig. 1:**
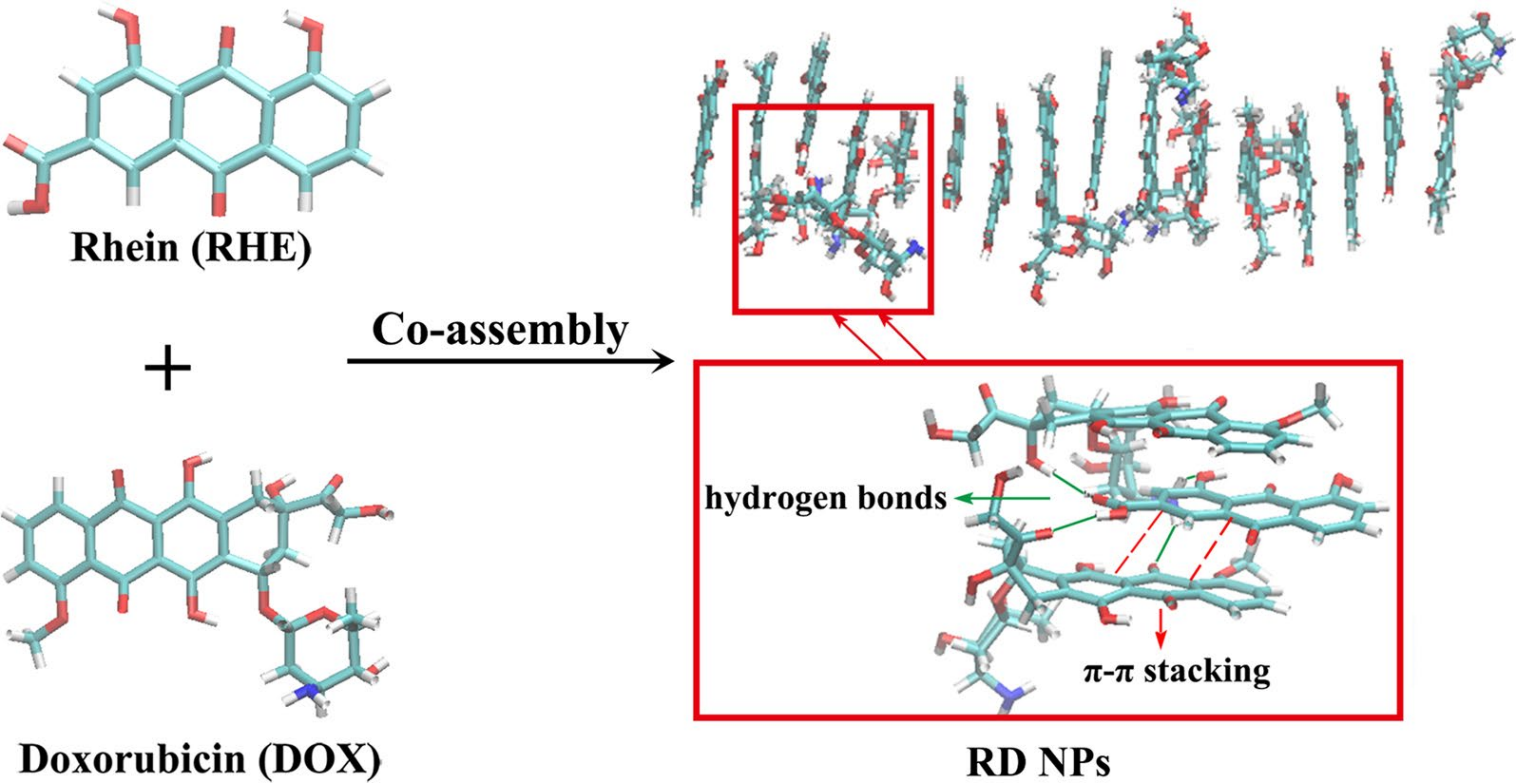
MD simulations of the co-assembly process for RHE and DOX molecules in water after 11 ns. The magnified graph shows representative structures that formed π-π interactions and hydrogen bond interactions

The structure of DOX shares common features with a surfactant, namely, unsaturated anthracycline rings and a saturated end of the ring system that function as the hydrophobic part of the molecule or the hydrophilic portion, respectively, and abundant hydroxyl groups are present adjacent to the amino sugar. Since RHE is hydrophobic, DOX might potentially be designed to solubilize and nanosize RHE [25]. Meanwhile, the anticancer effect of DOX might be hampered by the reactivation of the NF-κB pathway during the migration and invasion of tumor cells by upregulating the expression of target genes. On the other hand, RHE is able to suppress the transcriptional activity of NF-κB by inhibiting the expression of MMP-9. Therefore, the combination of DOX and RHE is hypothesized to exert a synergistic therapeutic effect on metastatic cancer [9]. The novel and green approach to the formulation of excipient-free nanodispersions has therefore been developed using the self-assembly of RHE and DOX molecules that was associated with interaction forces (hydrogen bond interactions, π-π stacking interactions and hydrophobic interactions) and did not involve any organic solvents (Fig. 1). The strong interactions between DOX and RHE form a hydrophobic core, leaving the hydrophilic daunosamine heads of DOX unbound and therefore the nanoparticle becomes dispersible.

Compared to other nanoparticles [31–33], amphiphilic small molecules were designed as stabilizers, and the strong interactions between RHE and DOX were applied to develop nanoparticles with “green” procedures. The combination of DOX and RHE potentially exerts synergistic antiproliferative effects on metastatic breast cancer, with fewer side effects. With the advantage of nanosizing, RD NPs improved the intracellular retention of DOX. A cell scratch assay indicated that RD NPs inhibited the metastasis of 4T1 cells. Compared to free DOX, the combination of DOX and RHE in nanoparticles showed a stronger ability to induce cell apoptosis. Hence, RD NPs not only suppressed primary tumor growth and inhibited tumor metastasis but also reduced the side effects of DOX, which also provide inspiration for the fabrication of new excipient-free nanodispersions, particularly for small molecular antitumor agents.

## Experimental section

### Materials

DOX (purity 98%) and RHE (purity 98%) were obtained from Shanghai Yuanye Biological Technology Co., Ltd. (Shanghai, China). MTT was purchased from Fluka (MO, USA). The Annexin V-FITC Kit was purchased from Nanjing Jiancheng Bioengineering Institute (Nanjing, China). All other reagents or chemicals used in the present study were analytical grade reagents and used without further treatment.

### Molecular dynamics (MD) simulations

Computer simulation provides another method for evaluating the self-assembly process of complex systems. Classical MD simulations were conducted using the AMBER16 package [34]. The force field parameters for DOX and RHE molecules were obtained from the generalized Amber force field (GAFF). The atomic charges were generated with the restrained electrostatic potential (RESP) method [35]. An artificial box containing 8 DOX, 8 RHE and 2000 water molecules was generated using the leap module in Amber Tools. Periodic boundary conditions were used, with the long-range electrostatic interactions computed with the particle-mesh Ewald summation. The SHAKE algorithm was used to constrain the bonds involving hydrogen atoms that did not stretch. The cutoff value for the nonbonded interactions was 8.0 Å. The system was optimized for 2500 steps with the steepest descent method followed by 2500 steps with the conjugate gradient method. Then, the system was gradually heated from 0 K to 300 K under the NVT ensemble for 200 ps. Finally, the MD trajectory was accumulated for 12 ns under the NPT ensemble (300 K and 1 atm) [24].

### Preparation of nanoparticles

RD NPs were developed using a “green self-assembly approach” [12]. Briefly, 30 μL of the RHE (20 mM) solution was gradually injected into 3 mL of deionized water (60 °C) under ultrasonication (ultrasonic cleaner, KH-500DE, Kunshan, China, 300 W). Then, 60 μL of DOX (10 mM) was added, followed by 1 h of ultrasonication. Afterwards, the resulting nanodispersions were ultrafiltered to completely remove the free drugs. The RD NPs were finally prepared for further use. Other formulations with various molar ratios (feeding ratio) of RHE to DOX were prepared as described above. Equal volumes of the solution of RHE (2 mg/mL) in 40% polyethylene glycol (PEG 400) (containing 5% NaHCO_3_, m/v) and the aqueous DOX solution (2 mg/mL) were mixed to prepare the mixture of RHE and DOX (RHE/DOX).

### Characterization of nanoparticles

The diameter and zeta potential of RD NPs were characterized using dynamic light scattering (DLS) with a Malvern Zeta-sizer (NanoZS90, Malvern Instruments Ltd., UK). Transmission electron microscopy (TEM) was performed using a Zeiss EM 912 Omega TEM at an acceleration voltage of 120 kV. Scanning electron microscopy (SEM) was performed using a Quanta FEG 250 SEM at an acceleration voltage of 20 kV. A fluorescence spectrometer (Hitachi F-4500) was applied to acquire the spectra of free RHE, free DOX and RD NPs (λex (RHE) = 360 nm; λex (DOX) = 475 nm). The concentrations of RHE and DOX in the nanoparticles were determined using HPLC (Waters Alliance 2695 System, Milford, America). For chromatography, a Kromasil C18 column, acetonitrile/sodium dodecyl sulfate solution, and a 254-nm detection wavelength were used [36, 37]. The encapsulation efficiency (EE) was detected using an ultrafiltration approach. The free drug in the filtrate was detected, and the EE was calculated using the following equation:

1 $$ {\text{EE }}\left( \% \right)\, = \,\left( {{\text{total drug}}{-}{\text{free drug}}} \right)/{\text{total drug}}\, \times \, 100. $$

The storage stability of RD NPs was evaluated at 4 °C and room temperature. The diameter and polydispersity index (PDI) were detected at predetermined time points. For the serum stability study, RD NPs were incubated with fetal bovine serum (FBS) at a volume ratio of 2:1. The diameter and PDI were analyzed at designated time points (0, 1, 2, 4, 8, 12, and 24 h).

### Drug release test

A dialysis method was applied to detect the in vitro release profile [3]. RHE, DOX and RD NPs were sealed in dialysis bags (3500 Da) and immersed in phosphate-buffered saline (PBS) solutions (pH 7.4 or 5.0). The tubes were gently shaken in a water bath. At specific time points, 1 mL of the external buffer was withdrawn and immediately replaced with 1 mL of fresh media, and the amounts of RHE and DOX released were analyzed using HPLC, as mentioned above. All experiments were performed in triplicate.

### Cell culture

The 4T1 mouse breast cancer cell line was obtained from the Shanghai Institute of Biochemistry and Cell Biology (Shanghai, China). 4T1 cells were incubated with Dulbecco’s Modified Eagle’s Medium (DMEM) in an atmosphere containing 5% CO_2_ at 37 °C.

### Cellular uptake analysis

Ten thousand 4T1 cells were seeded for analysis using a confocal laser scanning microscope (CLSM, Carl Zeiss AG, Germany). Cells were treated with RHE, DOX, RHE/DOX and RD NPs (2 μM RHE and 1 μM DOX) for 4 h. Cells were visualized with a CLSM. RHE was excited at a wavelength of 360 nm, and DOX was excited at a wavelength of 475 nm [38].

### In vitro cytotoxicity assay

The cytotoxicity of RHE, DOX, RHE/DOX and RD NPs toward 4T1 cells was evaluated using MTT assays [3, 23]. Briefly, cells were seeded and cultured. Then, increasing concentrations of RHE, DOX, RHE/DOX and RD NPs (5, 10, 20, 50 and 100 μM RHE; and 0.2, 0.4, 1, 2 and 5 μM DOX) were added to the medium in each well. After a 48-h incubation, MTT was added. Then, the absorbance was measured at 490 nm using a microplate reader. Cell viability was calculated using the following equation:

2 $$ {\text{Cell viability }}\left( \% \right)\, = \,{\text{O}}.{\text{D}}._{{({\text{sample}})}} /{\text{O}}.{\text{D}}._{{({\text{control}})}} \, \times \, 100 $$

The combination index (CI) of RHE and DOX was analyzed using the Chou − Talalay equation [39]:

3 $$ {\text{CI}}x\, = \,\left( {\text{D}} \right) 1/\left( {{\text{D}}x} \right) 1\, + \,\left( {\text{D}} \right) 2/\left( {{\text{D}}x} \right) 2 { }\left( 3\right) $$

D*x* represents the required dose of RHE or DOX for an *x*% inhibition rate, and (D)1 and (D)2 indicate the doses of RHE and DOX, respectively, achieving an inhibitory effect in combination at the same level. CI values less than 1 indicate synergism, CI values equal to 1 indicate an additive effect, and CI values greater than 1 represent an antagonistic effect.

### Apoptosis assay

Apoptosis in 4T1 cells exposed to RHE, DOX, RHE/DOX and RD NPs was measured using annexin-propidium iodide staining. Cells were treated with RHE, DOX, RHE/DOX and RD NPs (RHE-equivalent dose of 2 μM or a DOX-equivalent dose of 1 μM). The cells were collected and stained with annexin V-FITC/PI double staining after a 48 h incubation. Cellular apoptosis was quantitatively determined using flow cytometry. Untreated 4T1 cells served as a control.

### Cell scratch assay

After 4T1 cells (1.5 × 10^5^ cells per well) formed a confluent monolayer, the monolayer was scratched to produce a gap. Then, cells were incubated with RHE, DOX, RHE/DOX and RD NPs at a concentration equal to 2 μM of RHE and 1 μM of DOX for 48 h. Images of the scratches were captured at 0 and 48 h with the microscope and ImageJ software was used to quantify migration. Meanwhile, the areas detected at 48 h were normalized to the initial areas to determine the relative migration rates [26, 40].

### In vitro migration and invasion

4T1 cells (5 × 10^3^ cells per well) were added to the upper chambers of Transwell inserts. The cells were then treated with RHE, DOX, RHE/DOX and RD NPs (equal to 2 μM of RHE and 1 μM of DOX) for 48 h. For the invasion assays, 4T1 cells (5 × 10^3^ cells per well) were added to the upper chambers that had been coated with Matrigel. The cells were then treated with the drugs (equal to 2 μM of RHE and 1 μM of DOX) and incubated for 48 h. Cells that passed through the membrane and adhered to the lower surface were stained with crystal violet and were then quantified using a microplate reader [40].

### Western blot analysis of cells

4T1 cells were treated with RHE, DOX, RHE/DOX and RD NPs (2 μM RHE and 1 μM DOX) for 48 h. Total cellular proteins were extracted using a protein extraction kit. Protein concentrations were measured using a bicinchoninic protein assay. After proteins were separated on gels, transferred to a membrane and the membrane was blocked, the membrane was incubated with a primary antibody against NF-κB P65, MMP-9, Bcl-2, Bax or β-actin (Wuhan Servicebio Technology Co., Ltd., China; diluted at 1:300) overnight, and then the membrane was incubated with a secondary antibody. The resulting bands were visualized using an ECL-plus detection system [25].

### Animals

Female BALB/c mice (18–2 g) and male Sprague–Dawley rats (180–220 g) were purchased from the Shanghai Laboratory Animal Center (Shanghai, China). Animal assays were conducted in accordance with the Guidelines for Animal Experimentation of Nanjing University of Chinese Medicine, and the protocol was approved by the institution’s Animal Ethics Committee (Nanjing, China. Certificate No.: SYXK-2018-0049).

4T1 cells were suspended and injected into the right inguinal mammary fat pad of the mice to establish an orthotropic 4T1 tumor-bearing mouse model [41]. The tumor volume (V) was assessed by measuring the length (a) and width (b) with calipers and then calculated using the following equation:

4 $$ {\text{V}}\, = \,{\text{a}}\, \times \,{\text{b}}^{ 2} / 2. $$

### Pharmacokinetic and biodistribution analyses

Sprague–Dawley rats (n = 6) were injected with RHE, DOX, RHE/DOX and RD NPs at an RHE dose of 5 mg/kg and DOX dose of 5 mg/kg via the tail vein to determine the pharmacokinetic profiles of RD NPs. At 0.083, 0.167, 0.25, 0.5, 1, 2, 4, 6, 8, 12 and 24 h post-injection, 500 µL of blood were collected and centrifuged to obtain the plasma. Plasma samples (100 µL) were mixed with 500 μL of ethyl acetate, 10 μL of 5 μg/mL ibuprofen and 0.5 μg/mL daunorubicin (used as internal standards for RHE and DOX, respectively), and 10 µL of 10% acetic acid. The mixture was vortexed and then centrifuged. The organic layer was evaporated to dryness under a nitrogen stream and reconstituted with methanol. An aliquot (10 μL) was subjected to ultra-performance liquid chromatography-tandem mass spectrometry (UPLC-MS/MS) [40].

When the tumor size reached approximately 500 mm^3^, 4T1 tumor-bearing mice were intravenously injected with DOX and RD NPs (5 mg/kg of DOX). For ex vivo imaging, the mice were sacrificed at 12 and 24 h after the injection. The main organs were harvested and immediately imaged after sacrifice with an in vivo imaging system using the DOX channel. The semiquantitative analysis of the biodistribution of the average fluorescence intensity was determined using Image Lab Software. Additionally, the harvested tumors were frozen, sectioned, and then observed using a CLSM.

For the biodistribution analysis, 4T1 tumor-bearing mice were injected with RHE, DOX, RHE/DOX and RD NPs at an RHE dose of 5 mg/kg and a DOX dose of 5 mg/kg (3 mice per group at each time point). At 12 and 24 h after administration, the mice were sacrificed and the main tissues were harvested. Tissue samples were weighed and homogenized, and then the samples were processed and analyzed [12, 42].

### In vivo antitumor efficacy

BALB/c mice bearing 4T1 tumors were randomly divided into 5 groups (n = 5) when the tumor size reached approximately 100 mm^3^ to monitor the antitumor efficacy of RD NPs. Animals were treated saline, RHE, DOX, RHE/DOX and RD NPs (RHE at 5 mg/kg and DOX at 5 mg/kg) every other day via an intravenous (*i.v*.) injection. The tumor size and body weight were observed every other day, and the tumor inhibition rate (TIR) was calculated using the following equation:

5 $$ {\text{TIR }}\left( \% \right)\, = \,\left( {{\text{tumor weight}}_{{({\text{control group}})}} {-}{\text{tumor weight}}_{{({\text{sample group}})}} } \right)/{\text{tumor weight}}_{{({\text{control group}})}} \, \times \, 100. $$

The mice were sacrificed on day 12, and tumors were harvested and subjected to a pathological analysis (hematoxylin and eosin (H&E) and TUNEL detection). Each of the five pulmonary lobes was separated, and tumors on the surface were analyzed. Then, the lungs were sectioned and stained with H&E [43].

### Immunohistochemical staining

The expression of NF-κB P65, MMP-9, Bcl-2 and Bax was analyzed in tumor tissues using immunohistochemical staining. Tumor tissues were sectioned and deparaffinized using EZ-Dewax. The paraffin sections were first blocked to prevent nonspecific binding. Then, sections were incubated with primary antibodies. After an incubation with the avidin–biotin complex, the immunoreactivity was visualized [3].

### Western blot analysis of tissues

For the Western blot assay, tissues were homogenized. The total protein content was determined using a BCA protein assay kit. After proteins were separated on gels, transferred to membranes and the membranes were blocked, the membranes were incubated with primary antibodies against NF-κB P65, MMP-9, Bcl-2, Bax and β-actin. Next, the membranes were incubated with a secondary antibody. Then, the membranes were observed using an imaging system, and the densitometry analysis of the bands was performed with Image-Pro Plus software.

### Safety profiles

BALB/c mice were injected with saline, DOX and RD NPs (DOX at 5 mg/kg) every other day. On day 12, blood was collected and analyzed using a blood analyzer and autoanalyzer. The major organs were harvested for H&E staining.

### Statistical analysis

All data are presented as means ± standard deviations (SD). Data were compared between groups using one-way analysis of variance, followed by Student’s t-test. Significant differences are indicated as *p < 0.05, **p < 0.01, or ***p < 0.001.

## Results and discussion

### MD simulations

MD simulations were performed for RHE and DOX to help us understand how the molecules interact with each other in aqueous solutions. The RHE and DOX molecules were initially in a dispersed state. The π-π stacking interactions formed between RHE and DOX molecules at 0.5 ns; then these small-size clusters gradually accumulated and formed relatively large clusters at 2.0 ns. A stable aggregate was ultimately formed within 11 ns (Additional file 1: Figure S1).

Furthermore, the number of intermolecular hydrogen bonds and π-π stacking interactions between DOX and RHE molecules increased, indicating that RHE and DOX self-assembled in water to form RD NPs through π-π stacking and hydrogen interactions (Additional file 1: Figure S2). By analyzing the solvent-accessible surface areas (SASA) and the number of hydrogen bonds between the co-assembled structures and solvent water, hydrophobic interactions helped DOX and RHE molecules form the co-assembled structures (Additional file 1: Figure S3). Hence, hydrogen bond interactions, hydrophobic interactions and π-π stacking interactions would contribute to the co-assembly process.

In addition, the comparison to MD simulations of pure RHE and DOX alone in aqueous solution indicated that more π-π stacking and intermolecular hydrogen bonds formed between DOX and RHE molecules in RD NPs than in pure RHE and DOX alone (Additional file 1: Figure S4 and S5), suggesting that the driving force for self-assembly in individual drug molecules would be weaker than in the mixture. In the mixed system, DOX and RHE preferentially interacted to form RD NPs.

### Preparation and characterization of RD NPs

**Fig. 2:**
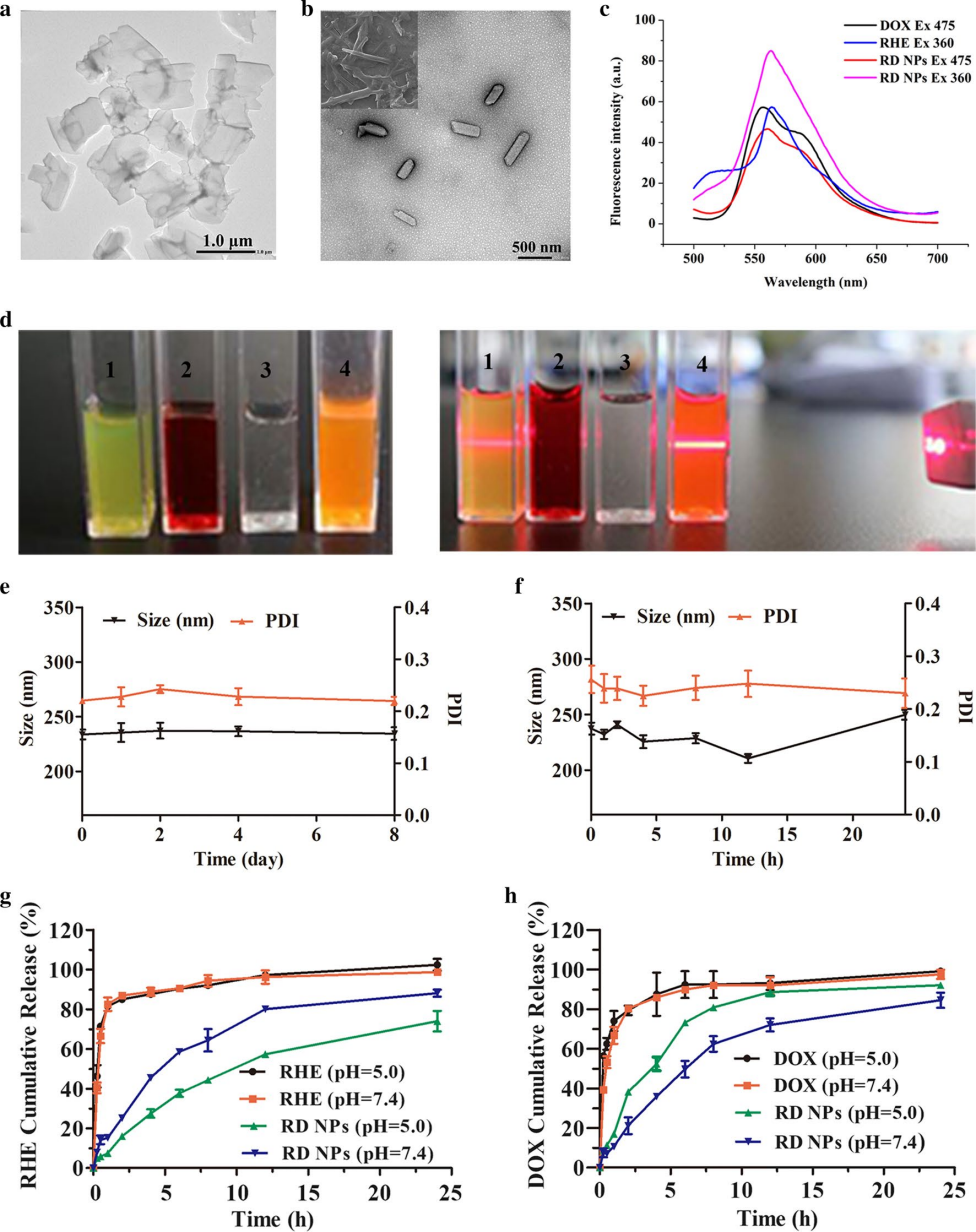
**a** TEM image of RHE. **b** TEM and SEM images of RD NPs. **c** Fluorescence spectra of RHE (200 μM), DOX (200 μM) and RD NPs (200 μM RHE and 200 μM DOX). λ_ex_ (RHE) = 360 nm; λ_ex_ (DOX) = 475 nm. **d** Photographs and the Tyndall effect of 1) RHE in a DMSO/water solution (99% water), 2) DOX in aqueous solution, 3) H_2_O, and 4) RD NPs in a DMSO/water solution (99% water). **e** The particle size and PDI of RD NPs during storage at 4 °C. **f** Changes in the particle size and PDI of RD NPs stored in 10% FBS for 24 h. Cumulative release of **g** RHE and **h** DOX from RHE, DOX and RD NPs at pH 7.4 and 5.0

The assembled RHE particles formed irregular sheet-like structures, which were characterized using TEM (Fig. 2a). When the DOX was added to the RHE suspension (RHE/DOX molar ratio = 1:1), DOX molecules coassembled with RHE via interaction forces and formed rod-like RD NPs (Fig. 2b) with diameters of approximately 240 nm (Additional file 1: Figure S6). DOX was clear and transparent in aqueous solution, and the solution of RD NPs showed obvious Tyndall light scattering (Fig. 2d). The DLS analysis of RD NPs revealed a narrow monomodal distribution with a small mean hydrodynamic diameter of 249.90 ± 5.20 nm and a PDI of 0.14 ± 0.03, which was within the accepted range for efficient EPR and ensured a passive tumor targeting effect. The surface charge of RD NPs was determined to be 25.67 ± 1.03 mV (Additional file 1: Figure S7). The EEs of RHE and DOX were 96.23 ± 0.22% and 50.98 ± 7.72%, respectively (Table 1). For comparison, the EEs of erlotinib and DOX were 50% and 93% in erlotinib/DOX codelivering nanoparticles, indicating that compared with other codelivering nanoparticles, the RD NPs exhibited an improved drug-carrying capacity [44]. Furthermore, the emission intensity of RD NPs was lower than the aqueous DOX solution due to the aggregation induced by π-π stacking interactions (Fig. 2c). RD NPs showed an increased emission intensity compared to RHE, indicating that the aggregation of RHE was partially inhibited by the interactions between RHE and DOX. This inhibition may be attributed to the encapsulation of RHE in the hydrophobic domains of DOX.

**Table 1:** The encapsulation efficiencies (EEs) of RHE and DOX in their self-assembled nanoparticles (NPs)

Molar ratio	RHE (%)	DOX (%)
RHE/DOX = 1:0.5	98.29 ± 0.58	70.26 ± 3.47
RHE/DOX = 1:1	96.23 ± 0.22	50.98 ± 7.72
RHE/DOX = 1:2	94.14 ± 2.51	30.54 ± 5.81

Colloid stability plays an important role in the biomedical application of nanoscale drug-delivery systems. No significant changes in the hydrodynamic diameter or the PDI of RD NPs were observed for up to 8 days at 4 °C (Fig. 2e). The diameter measured using DLS remained unchanged over 8 days, suggesting the excellent stability of RD NPs at room temperature (Additional file 1: Figure S8). Additionally, the coassembled NPs retained good colloidal stability in 10% FBS (Fig. 2f). Although no surfactants or excipients were applied, the RD NPs displayed desirable stability without any precipitation and phase separation.

RHE, DOX, and RD NPs were dispersed in buffer solutions at pH 7.4 and 5.0 to measure the release profile at 37 °C for 24 h. In Fig. 2g and h, a significant difference in the release properties of free RHE and DOX was not observed at different pH values, and approximately 80% of the drugs was released during the first 2 h. In contrast, the release rate was slower in the RD NPs than in the free RHE and DOX solutions. Approximately 25% of RHE and 20% of DOX were released from RD NPs (pH = 7.4) in 2 h. A total of 73.95 ± 8.93% of RHE and 92.07 ± 1.78% of DOX were released from NPs at pH 5.0 within 24 h; both release rates would improve the therapeutic efficacy. In contrast, approximately 62.70% of ursolic acid is released from ursolic acid NPs under the same conditions [45]. Moreover, RHE and DOX release from RD NPs were best modeled using a first-order kinetic model with R^2^ > 0.99 (Additional file 1: Table S1). The relatively slower RHE release rate might result from the carboxyl groups of RHE, which are more readily protonated under acidic conditions, decreasing its solubility and decelerating its diffusion. Similarly, the slightly faster release of DOX is potentially due to the increased solubility associated with the protonated amino group under acidic conditions [46].

### Cellular uptake analysis

**Fig. 3:**
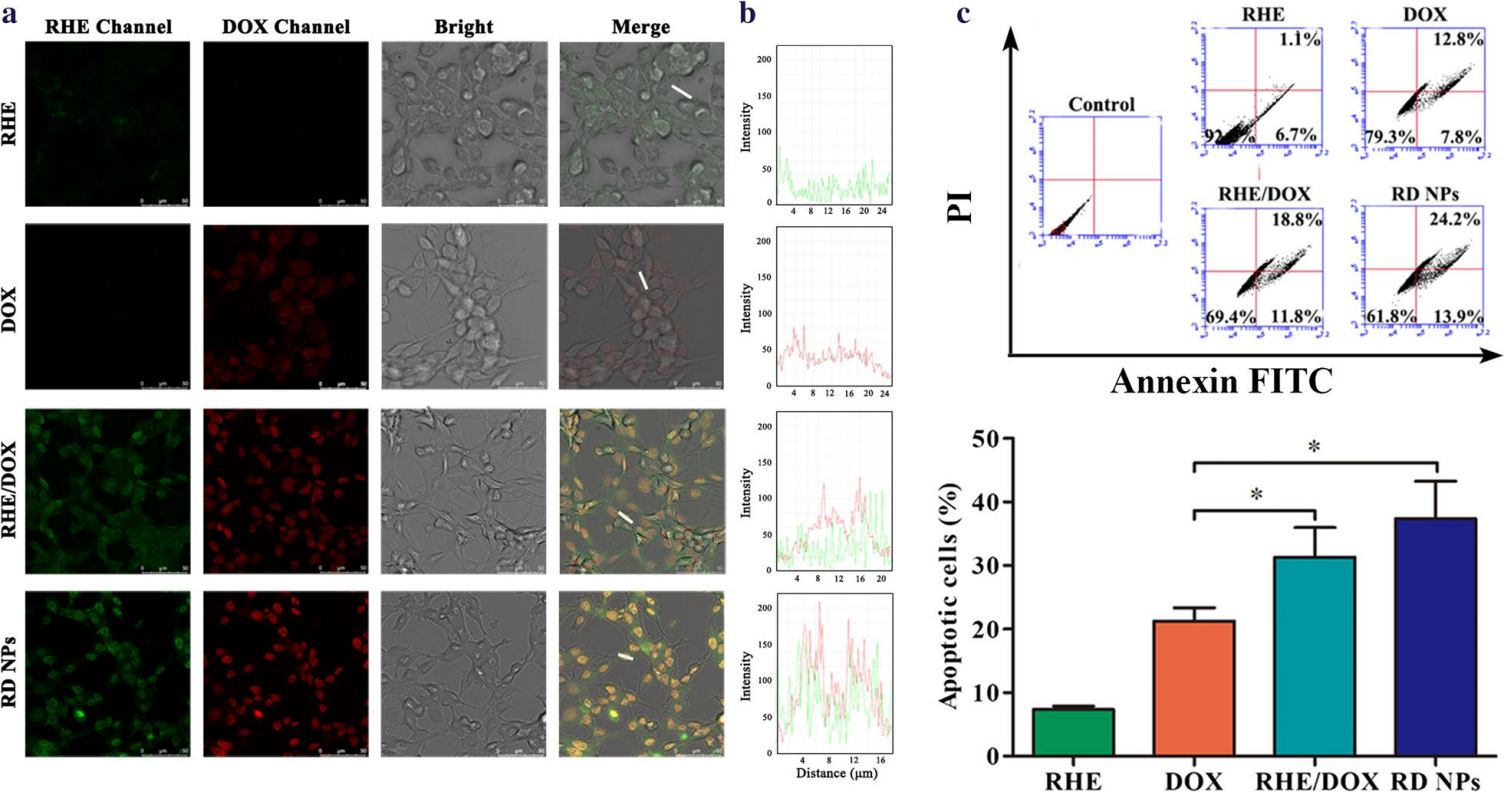
**a** CLSM images of the cellular uptake of RHE, DOX, RHE/DOX and RD NPs at equivalent RHE (2.0 μM) and DOX concentrations (1.0 μM) in 4T1 cells. **b** Line-scanning profiles of the fluorescence intensity of 4T1 cells incubated with these preparations. **c** Apoptosis of 4T1 cells after an incubation with RHE, DOX, RHE/DOX and RD NPs (n = 3, mean ± SD). *p < 0.05

We compared the cellular uptake of RHE, DOX, RHE/DOX and RD NPs by 4T1 breast cancer cells to verify that RD NPs were efficiently internalized by cancer cells. According to the CLSM images (Fig. 3a), RD NPs noticeably increased the cellular uptake of RHE and DOX, leading to much higher intensities of green and red fluorescence in 4T1 cells compared with the free RHE, free DOX and RHE/DOX groups. These data were also qualitatively corroborated by the line-scanning profiles of fluorescence intensity over selected cells (Fig. 3b). The green and red lines represent RHE and DOX fluorescence intensities, respectively. The fluorescence intensity of RHE and DOX in the RD NPs was much higher than the RHE, DOX or RHE/DOX groups, and the colocalization of red and green fluorescence showed that DOX and RHE were codelivered to the cells. The increased accumulation of these drugs in 4T1 cells following treatment with the nanosuspension was expected to improve their antitumor effect.

### In vitro antitumor efficacy

The in vitro cytotoxicity of RHE, DOX, RHE/DOX and RD NPs was assessed in 4T1 cells using MTT assays to evaluate the antitumor efficacy of RD NPs. As shown in Additional file 1: Figure S9A, B and C all formulations exerted dose-dependent effects on 4T1 cells. Free RHE and free DOX had IC_50_ values of 73.86 ± 4.40 and 1.30 ± 0.22 μM, respectively (Table 2). Compared with the single-drug treatments, combination therapy with RHE and DOX resulted in relatively lower cell viability. The IC_50_ of DOX was reduced to 0.84 ± 0.16 μM when combined with RHE in the mixture. Furthermore, the cell viability in the RD NP group was further reduced, with an IC_50_ of 0.63 ± 0.23 μM. The IC_50_ of PTPNs in 4T1 cells was 63-fold lower than that of free paclitaxel, and the IC_50_ of RD NPs was 73-fold lower than that of free RHE, indicating that RD NPs showed better antitumor efficiency than PTPNs [47]. The increased antitumor efficacy of RD NPs was potentially attributed to several factors. RHE and DOX were simultaneously internalized in the cells, which allowed both drugs to support the individual antitumor effects of the other drug. In addition, RD NPs were taken up more efficiently by cells than free drugs. In addition, we calculated the CI values of RHE/DOX and RD NPs. As shown in Table 2, the CI value of RHE/DOX was 0.68, indicating a strong synergistic antiproliferative effect. Moreover, RD NPs had a CI value of 0.51; therefore, the relatively profound synergistic effect of RD NPs was consistent with the cytotoxicity results.

**Table 2:** The IC_50_ values of RHE/DOX and RD NPs in 4T1 cells

Samples	IC_50_ (μM)	
	RHE	DOX
RHE	73.86 ± 4.40	–
DOX	–	1.30 ± 0.22
RHE/DOX	2.53 ± 0.43	0.84 ± 0.16
RD NPs	1.88 ± 0.60	0.63 ± 0.23

Apoptosis refers to a programmed cell death pathway controlled by genes that maintain the stability of the internal milieu. The apoptosis rate is an important index for evaluating the therapeutic effects of antineoplastic agents. We measured the apoptosis induced by RD NPs by performing double staining with annexin V-FITC and PI. As displayed in Fig. 3c, RHE/DOX and RD NPs showed a stronger ability to induce cell apoptosis (31.30 ± 4.69% and 37.40 ± 5.88%, respectively) than free DOX (21.27 ± 2.08%). Meanwhile, the RD NP group induced an even higher level of apoptosis than the RHE/DOX group, which was probably due to the increased cellular uptake of the NPs compared to the free drugs.

### In vitro anti-metastatic effects

**Fig. 4:**
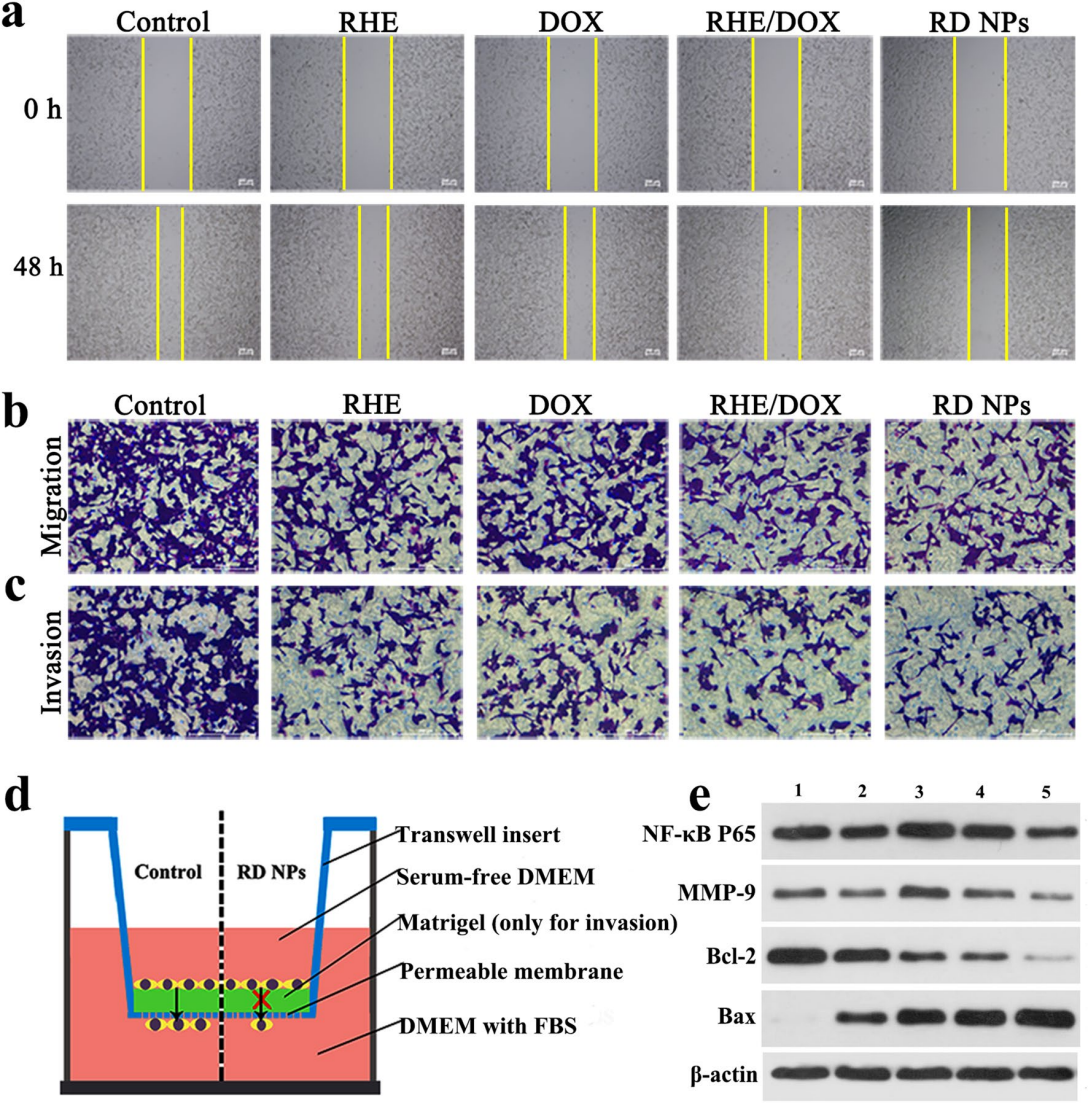
**a** The images of cells captured 48 h after scratching. Microscopy images of (**b**) the migration and (**c**) invasion of 4T1 cells that passed through the membrane after an incubation with RHE, DOX, RHE/DOX and RD NPs compared with the control group (untreated cells). **d** Schematic of 4T1 cell migration and invasion in the Transwell insert. **e** Western blots showing the levels of the NF-κB P65, MMP-9, Bcl-2 and Bax proteins in 4T1 cells. Lane 1: blank; Lane 2: RHE; Lane 3: DOX; Lane 4: RHE/DOX; and Lane 5: RD NPs

Next, the ability of RD NPs to inhibit the metastasis of 4T1 cells in vitro was detected. First, a cell scratch assay was applied to investigate cell migration, and the images of cells preincubated with various formulations were evaluated after scratching to evaluate the inhibitory effect of RD NPs on cell migration (Fig. 4a). As displayed in Fig. 4a and Additional file 1: Figure S10, the scratch healing rates of cells treated with RHE, DOX, RHE/DOX and RD NPs were 39.64 ± 5.84%, 53.66 ± 2.57%, 35.29 ± 3.22% and 28.32 ± 3.84%, respectively. Thus, RHE/DOX and RD NPs exerted a stronger inhibitory effect on the scratch healing rates than DOX. Moreover, migration and invasion assays (Fig. 4d) were performed to further determine the inhibitory effects of RHE, DOX, RHE/DOX and RD NPs on 4T1 cell migration. As exhibited in Fig. 4b and Additional file 1: Figure S11A, the cells treated with RHE, DOX, RHE/DOX and RD NPs exhibited decreased migration compared to the control, with migration rates of 53.05 ± 8.47%, 61.22 ± 2.08%, 47.42 ± 3.21% and 40.08 ± 8.54%, respectively, indicating that RHE/DOX and RD NPs exerted stronger inhibitory effects on migration than free DOX. Furthermore, the results of the invasion assay were consistent with the data obtained from the scratch healing and migration assays (Fig. 4c and Additional file 1: Figure S11B).

The inhibition of cell migration by RHE/DOX and RD NPs might be attributed to a decrease in the level of the NF-κB protein, which is an essential contributor to metastasis. NF-κB is a critical intermediate involved in the progression of cell migration, invasion and proliferation; thus, the NF-κB protein plays relatively important roles in cell metastasis and apoptosis. Therefore, the expression of a variety of proteins, including NF-κB P65, the metastasis-related protein MMP-9, the proapoptotic protein Bax and the antiapoptotic protein Bcl-2, were analyzed in 4T1 cells treated with RD NPs using Western blotting to detect the mechanisms by which RHE/DOX and RD NPs inhibit cell migration. As shown in Fig. 4e and Additional file 1: Figure S12, after treatment with RHE/DOX or RD NPs, the levels of NF-κB P65 and MMP-9 were significantly decreased compared with those of cells treated with DOX alone (*p* < 0.01). Furthermore, compared with the DOX group, the RD NP group presented significantly decreased Bcl-2 levels (*p *< 0.001). In addition, Bax levels were significantly increased in the RHE/DOX and DOX groups (*p *< 0.01). Thus, the anti-metastasis and apoptosis mechanism of the combination of RHE and DOX included the inhibition of NF-κB P65. Moreover, the RD NP group exhibited the lowest level of NF-κB P65 due to the increased cellular uptake of the NPs in this group compared to the uptake of drugs in the other groups.

### Pharmacokinetic and biodistribution analyses

**Fig. 5:**
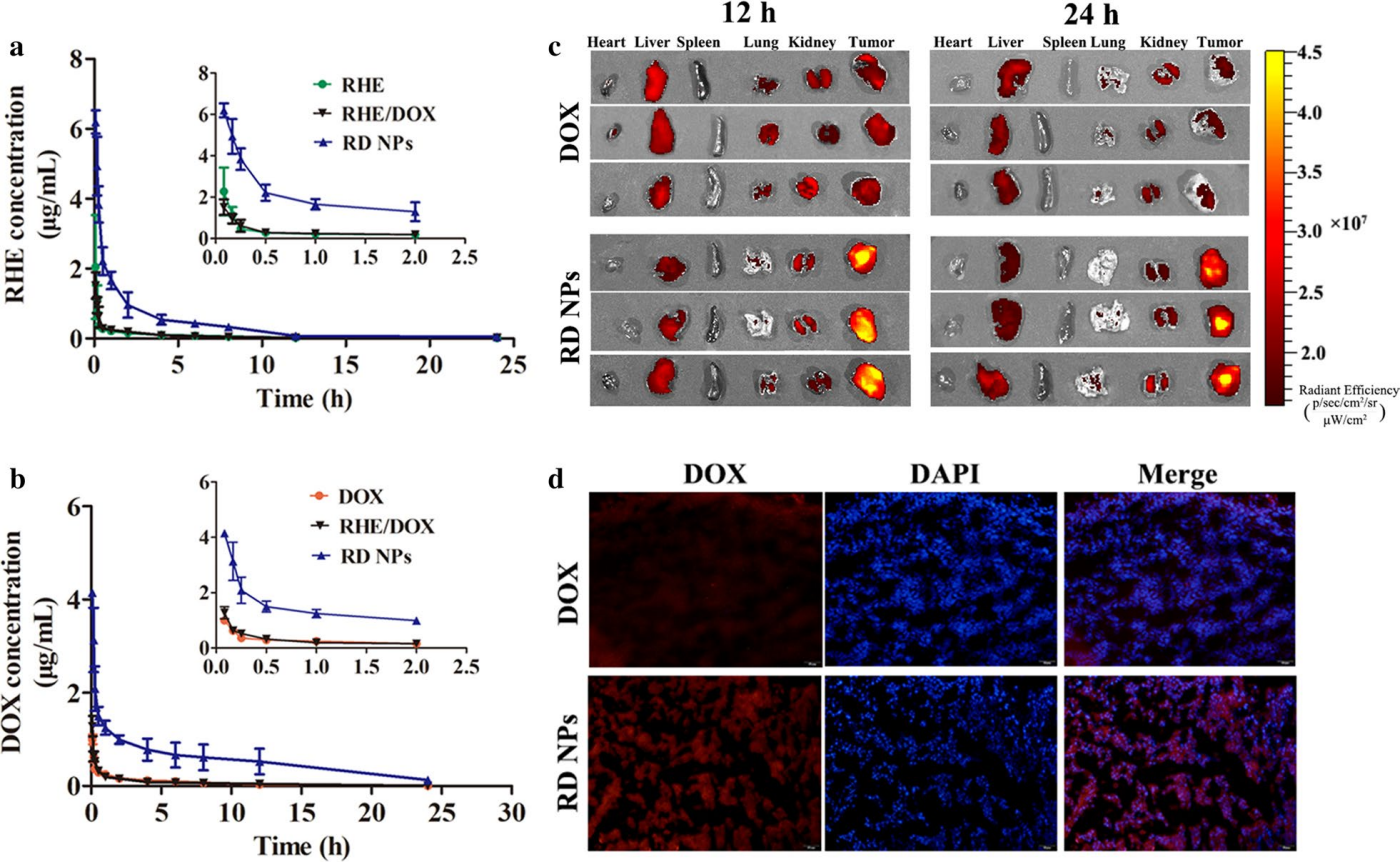
**a** The plasma RHE concentration–time curves of injected RHE, RHE/DOX and RD NPs (n = 6, mean ± SD). **b** The plasma DOX concentration–time curves of injected DOX, RHE/DOX and RD NPs (n = 6, mean ± SD). **c** *Ex vivo* images of tumors and major organs at 12 h and 24 h post-injection. **d** Confocal images of frozen tumor sections collected after 24 h. DOX is shown in red, and the nucleus is shown in blue (DAPI)

The pharmacokinetic behaviors of RHE and DOX were investigated after the intravenous injection of the three formulations. As shown in Fig. 5a, b, RD NPs exhibited a prolonged circulation time compared to RHE, DOX and RHE/DOX after an *i.v*. injection. The plasma concentrations of free RHE, free DOX or RHE/DOX decreased rapidly within 12 h, while RD NPs exhibited a significantly delayed blood clearance. The analysis of the pharmacokinetic parameters revealed that RD NPs increased the half-life of RHE from 1.81 h to 6.87 h (Table 3). Similarly, the RD NPs extended the half-life of DOX from 2.92 h to 7.14 h. While erlotinib/DOX codelivering nanoparticles extended the half-life of DOX from 1.81 h to 3.19 h [44], the use of RD NPs as anticancer codelivering nanoparticles is advantageous over erlotinib/DOX codelivering nanoparticles in prolonging the circulation time. In addition, the area under the curve (*AUC*_*0*-*∞*_) of RHE increased by ~ 11.36-fold for RD NPs compared to free RHE. Similarly, the *AUC*_*0*-*∞*_ of DOX increased by ~ 9.44-fold for RD NPs compared to free DOX. Based on these results, RD NPs showed improved pharmacokinetic profiles and might exhibit superior synergistic antitumor efficacy in vivo.

**Table 3:** Main pharmacokinetic parameters of drugs in rats after the intravenous injection of different drugs (n = 5)

Drugs	Formulations	*C*_max_ (μg/mL)	*T*_1/2_ (h)	*AUC*_0-∞_ (mg/mL·h)	*CL* (L/h/kg)	*MRT*_0-∞_ (h)
RHE	Free RHE	0.35 ± 0.10^***^	1.81 ± 0.57^*^	0.94 ± 0.52^***^	6.25 ± 2.65^*^	2.51 ± 0.98
	RHE/DOX	0.62 ± 0.29^***^	2.15 ± 0.10^*^	1.25 ± 0.52^***^	4.49 ± 1.86^*^	2.59 ± 1.07
	RD NPs	5.46 ± 0.92	6.87 ± 2.11	10.68 ± 1.04	0.47 ± 0.04	5.20 ± 2.31
DOX	Free DOX	0.36 ± 0.04^*^	2.92 ± 0.14^*^	1.59 ± 0.06^*^	3.15 ± 0.12^***^	5.07 ± 0.29^*^
	RHE/DOX	0.52 ± 0.07	3.21 ± 0.05^*^	1.61 ± 0.15^*^	3.11 ± 0.30^***^	5.24 ± 0.60^*^
	RD NPs	2.09 ± 0.48	7.14 ± 1.42	15.01 ± 5.55	0.37 ± 0.14	9.63 ± 2.33

^*^p < 0.05, ^**^p < 0.01, and ^***^p < 0.001 compared to RD NPs

Encouraged by the increased therapeutic efficiency of RD NPs in vitro, the biodistribution of RD NPs was detected using fluorescence imaging. Since the DOX fluorescence was weak in vivo, the mice were euthanized and tissues (heart, liver, spleen, lung, kidney and tumor) were removed for ex vivo imaging, which is a more accurate qualitative biodistribution analysis. As shown in Fig. 5c and Additional file 1: Figure S13, the quantitative analysis of DOX fluorescence in regions of interest ex vivo substantiated the superior tumor accumulation of RD NPs, as a higher DOX intensity was observed in the tumor at levels 1.68- and 3.38-fold higher than free DOX at 12 and 24 h, respectively. The intratumor distribution of DOX was also observed in frozen tissue sections at 24 h postinjection (Fig. 5d), which showed that treatment with RD NPs led to a higher DOX intensity in the tumor than the free DOX treatment. The drastically higher accumulation of RD NPs was probably due to the long-term circulation and the EPR effect of the NPs.

We conducted biodistribution experiments and performed a quantitative analysis to more intuitively and accurately observe the accumulation of DOX and RHE. Biodistribution assays were performed with RHE, DOX and RD NPs to evaluate the tumor accumulation of RHE and DOX. Free RHE accumulated at lower levels in tumor tissues than RD NPs (Additional file 1: Figure S14). Similarly, lower levels of DOX accumulated in tumor tissues from mice treated with free DOX and RHE/DOX than in mice treated with RD NPs. Moreover, 1.45-fold more RHE accumulated in tumors after treatment with RD NPs than free RHE at 24 h postinjection. Similarly, 3.42-fold more DOX accumulated in the tumors after treatment with RD NPs than free DOX at 24 h postinjection. In addition, the highest DOX concentration that accumulated at the tumor site in mice treated with RD NPs (10.94 ± 0.91 μg/g) was observed at 12 h, consistent with the data from the imaging of isolated tumors. Accordingly, RD NPs displayed higher tumor accumulation than the free drugs. Thus, RD NPs were efficiently delivered to the tumor via EPR effects. Moreover, RD NPs resulted in a low level of DOX accumulation in the heart. However, few NPs accumulated in other organs. This accumulation is a common biological challenge, as most NPs become rapidly sequestered from the blood, followed by their accumulation in organs of the reticuloendothelial system (RES), such as the spleen or lung [48].

### In vivo antitumor efficacy

**Fig. 6:**
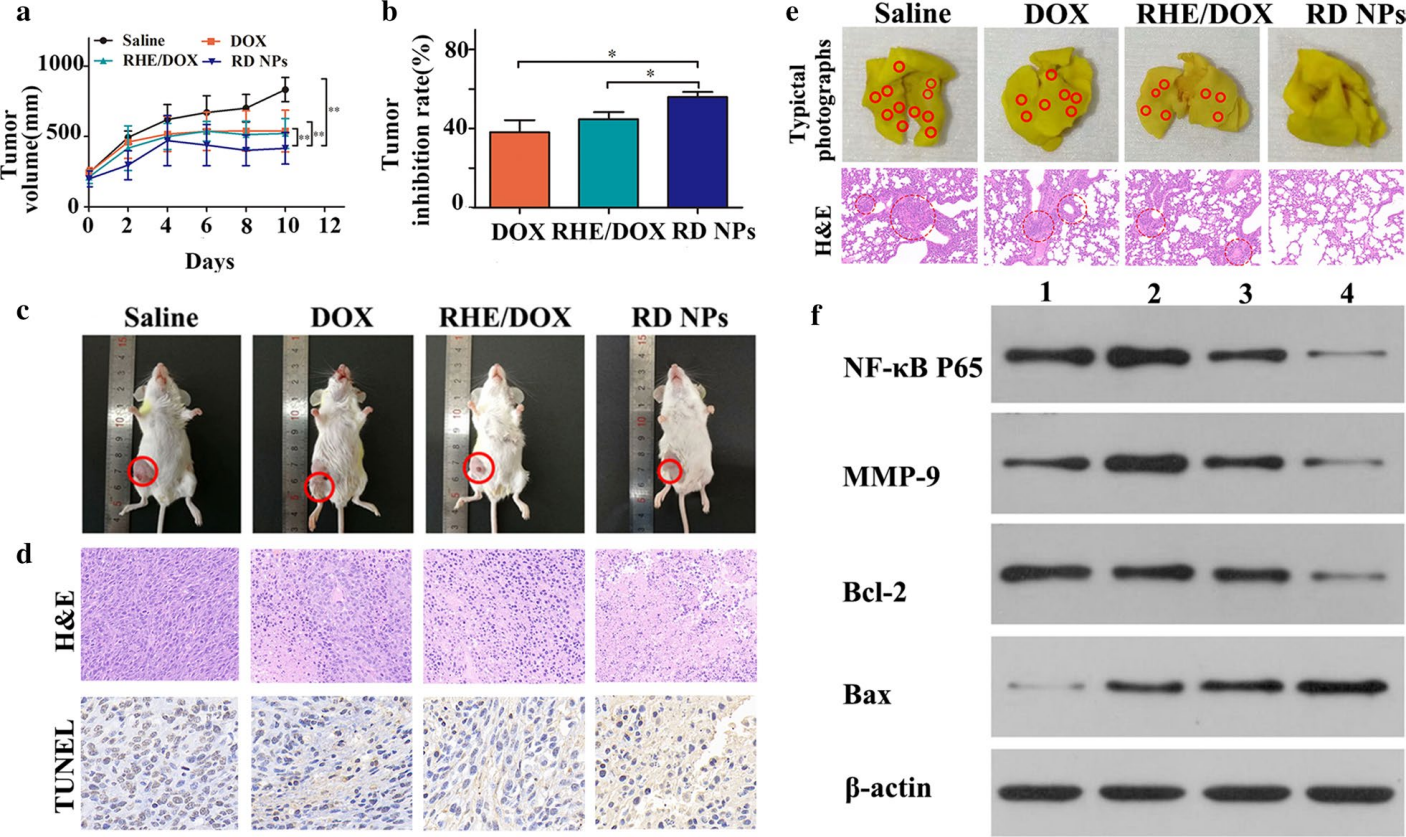
**a** 4T1 tumor growth curves of the study groups (n = 5, mean ± SD), including saline, DOX, RHE/DOX and RD NPs with an equivalent DOX concentration of 5 mg/kg. **b** Tumor inhibition in the study groups (n = 5, mean ± SD). **c** Representative images of the tumor-bearing mice captured 2 days after the final treatment. **d** Histological comparison of the tumor tissues at the conclusion of the study. **e** Representative photographs of whole lungs from mice in different groups and H&E staining of lung tissue sections. Surface lung metastatic lesions are denoted by red circles. **f** Western blots showing levels of the NF-κB P65, MMP-9, Bcl-2 and Bax proteins in tumors. Lane 1: blank; Lane 2: DOX; Lane 3: RHE/DOX; and Lane 4: RD NPs

A metastatic orthotropic 4T1 mammary adenocarcinoma model was established to evaluate the benefits of combination therapy with RD NPs. As shown in Fig. 6a, rapid tumor growth was detected in the saline group, while moderately restricted tumor growth was observed in animals treated with DOX or RHE/DOX. Tumor growth was significantly inhibited in the RD NP group, and the TIR was 55.90 ± 2.58% on day 10 (Fig. 6b), which was further confirmed by images of tumor xenografts in mice (Fig. 6c). The RD NP group displayed the lowest tumor volume after the final injection, indicating that the combination of RHE and DOX exerted a synergistic antitumor effect [49]. H&E staining of the tumor tissues harvested at the end of the study revealed the greatest cancer cell clearance in the RD NP group, which included coagulative necrosis and empty intercellular spaces, further validating the high antitumor activity of this treatment. TUNEL staining revealed the highest level of induced cell apoptosis in animals treated with this combination (Fig. 6d).

The orthotropic 4T1 tumors established in this therapeutic study spontaneously form lung metastases. We therefore evaluated whether RD NPs exerted an antimetastatic effect. Photographs of lung metastases and the H&E staining of lung sections were examined to assess the antimetastatic efficacy. As shown in Fig. 6e and Additional file 1: Figure S15, the RD NP treatment led to a significantly decreased number of metastatic lesions on the lung surface compared to the control treatment. In addition, the analysis of the lung tissue sections further supported these results. Thus, the RD NPs not only effectively inhibited primary tumor growth but also successfully suppressed tumor metastasis, consistent with the results of in vitro experiments showing antimetastatic effects [50].

Immunohistochemical staining was performed to determine whether tumor growth and aggressiveness were associated with decreased NF-κB expression (Additional file 1: Figure S16). Excised tumor sections from the RHE/DOX or RD NP groups displayed lower expression of the NF-κB P65 and MMP-9 proteins than the DOX group. Additionally, the highest expression of the proapoptotic protein Bax and the lowest expression of the antiapoptotic protein Bcl-2 was observed in the RD NPs group. Compared with free DOX, the efficient silencing of NF-κB P65 and the increased apoptosis induced by RHE/DOX were probably due to the presence of RHE. The RD NPs likely caused the greatest downregulation of NF-κB P65 and the highest apoptosis among the treatments because of the EPR effect on the RD NPs group. In addition, the improved pharmacokinetic profiles in the RD NPs group may have prolonged their circulation time and increased the accumulation of the drugs in the tumor.

NF-κB has been reported to play an important role in cancer cell migration and invasion. Thus, we investigated the effects of RD NPs on cell migration and invasion. The RD NP treatment groups exhibited significantly inhibited cell migration compared to the DOX treatment group (*p *< 0.001). As displayed in Fig. 6f and Additional file 1: Figure S17, the expression of MMP-9, which regulates cell invasion, was reduced in the RD NP treatment group compared with the DOX group (*p* < 0.001). Therefore, the increased inhibition of cell migration and invasion likely resulted from the greater inhibition of NF-κB activity and the reduced expression of MMP-9. Additionally, the highest level of the proapoptotic protein Bax was observed in the RD NPs group. Meanwhile, the lowest level of the antiapoptotic protein Bcl-2 was observed in the RD NPs group. Therefore, the inhibition of NF-κB or anti-NF-κB therapy might be applied as a possible therapeutic approach to control tumor metastasis. Therefore, the RD NPs would efficiently inhibit metastatic breast cancer.

### Safety profiles

**Fig. 7:**
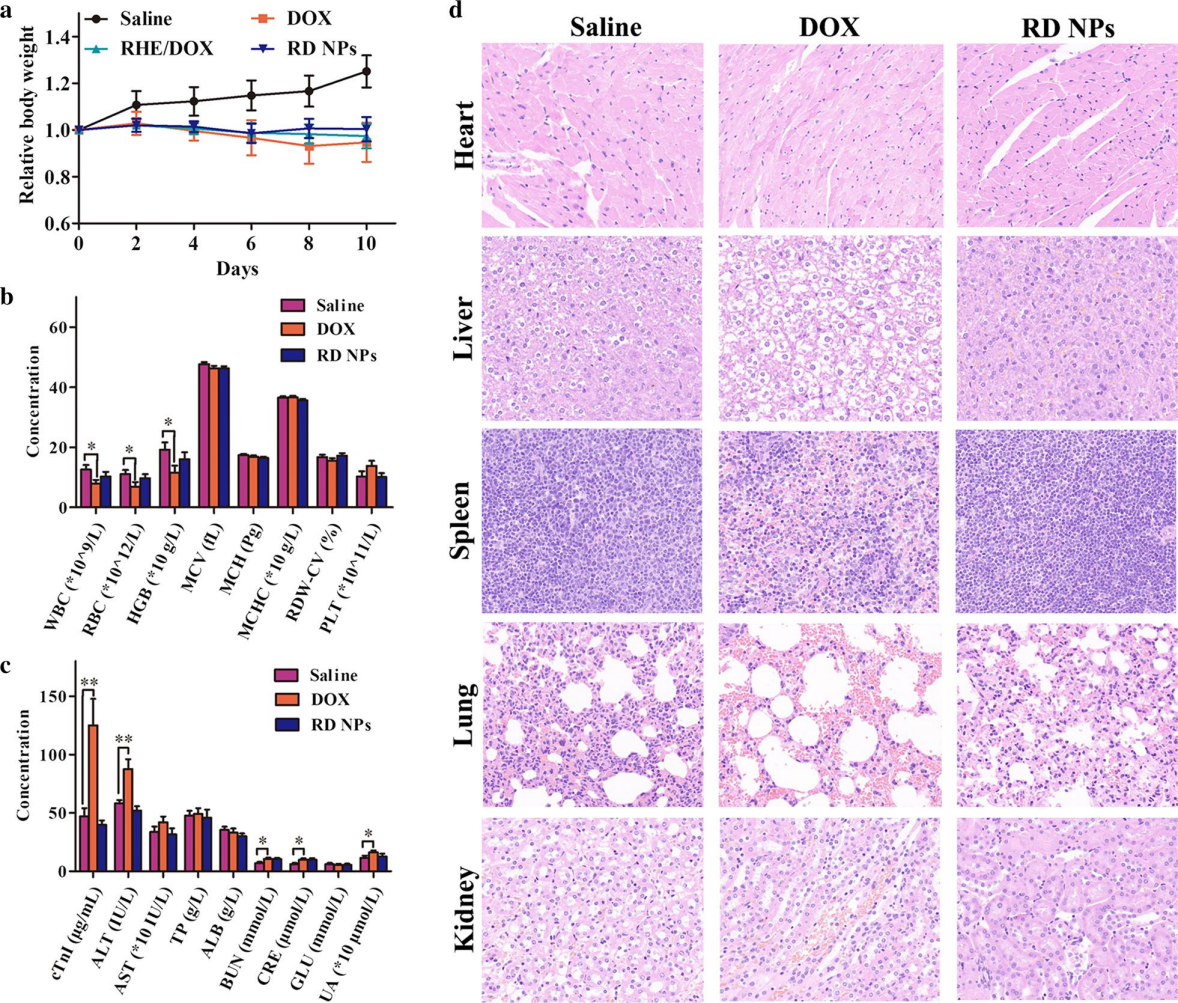
**a** Body weight curve of mice bearing tumors generated from 4T1 cells. **b** Hematological and (**c**) blood biochemical analyses of the mice. **d** Histopathology of tissues from the saline, DOX, and RD NPs groups (n = 5, mean ± SD). *p < 0.05 and ** *p *< 0.01

Body weight, biochemical functions and histopathological changes were evaluated and compared with saline and free DOX, which served as the negative and positive controls, respectively, to evaluate whether RD NPs induced any adverse effects during treatment. None of the treatments led to substantial body weight losses (Fig. 7a), indicating that RD NPs did not induce severe systemic toxicity. As shown in Fig. 7b, RD NPs did not exert a measurable adverse effect on blood cells or on the heart, liver and renal functions, based on the safety profiles. The numbers of peripheral blood cells were all within the normal ranges, indicating that no illnesses occurred, including hemolytic anemia and acute infection. In the blood chemistry analysis, the levels of cardiac troponin I (cTnI), the liver function biomarkers, e.g., alanine aminotransferase (ALT), aminotransferase, total protein and albumin, and the renal function biomarkers, e.g., blood urea nitrogen (BUN), creatinine (CRE), glutamic acid and uric acid (UA), were all normal, indicating that the RD NPs induced negligible hepatotoxicity and nephrotoxicity (Fig. 7c). In contrast, the levels of cTnI, ALT, BUN, CRE and UA were significantly increased in the free DOX group compared to the control group, indicating the presence of acute inflammation in the heart, liver and kidney. The histopathological results also verified these conclusions (Fig. 7d). The mice in the RD NPs and saline groups did not exhibit toxicity in the major organs, while an abnormal architecture was observed in the heart, liver and kidney tissues of animals pretreated with DOX, such as cavities in the heart, cytoplasmic degeneration of hepatocytes in the liver, and focal tubular necrosis in the kidney, indicating the apparent cardiotoxicity, hepatotoxicity and nephrotoxicity of DOX. In summary, RD NPs displayed superior therapeutic efficacy when administered at a safe level.

## Conclusions

Collectively, RHE was nanosized with the assistance of DOX to fabricate carrier-free RD NPs using a simple “green” preparation method. RHE and DOX molecules tended to coassemble via interaction forces (hydrogen bond interactions, π-π stacking interactions and hydrophobic interactions) and formed a rod-like morphology with satisfactory stability. Cellular uptake assays revealed that drug accumulation was significantly increased after treatment with RD NPs. Importantly, compared with free RHE, free DOX, and RHE/DOX, RD NPs exhibited superior cytotoxicity toward 4T1 cells, resulting in a potent synergistic therapeutic effect of the nanodispersions. The mechanistic study revealed that RD NPs inhibited the migration and invasion of tumor cells by reducing the expression of NF-κB and MMP-9. Taken together, the design of pure nanomedicines might represent a novel approach and inspire the fabrication of novel carrier-free nanodrugs for theranostics, particularly for achieving the aim of combination antitumor therapy with a synergistic effect.

## Supplementary information

**Additional file 1: Figure S1.** The process of RHE and DOX co-assembly in an aqueous solution extracted from the MD simulation. **Figure S2.** (A) The numbers of intermolecular hydrogen bonds and (B) π-π stacking interactions between DOX and RHE molecules during the MD simulation. **Figure S3.** (A) The SASA of the assembled clusters and (B) the number of hydrogen bonds between the clusters and solvent water during the MD simulation. **Figure S4.** The assembly of clusters of pure DOX (left panel) and pure RHE (right panel) in aqueous solution after an 11-ns MD simulation. **Figure S5.** Numbers of π-π stacking interactions and intermolecular hydrogen bonds formed in the co-assembled clusters of DOX and RHE, and self-assembled clusters of DOX and RHE alone, respectively. **Figure S6.** The size of RD NPs. **Figure S7**. The zeta potential of RD NPs. **Figure S8**. The particle size and PDI of RD NPs during storage at room temperature. **Figure S9.** *In vitro* cytotoxicity of 4T1 cells treated with (A) RHE, (B) DOX, (C) RHE/DOX and RD NPs for 48 h. **Figure S10.** Quantitative analysis of the scratch healing rate based on the images shown in Fig. 4A. **Figure S11.** Quantitative analysis of migrating or invading cells based on the images shown in Fig. 4B and 4C. **Figure S12.** Quantitative analysis of the relative intensities of the NF-κB P65, MMP-9, Bcl-2 and Bax bands based on the images shown in Fig. 4E. **Figure S13.** Semiquantitative analysis of the *ex vivo* DOX fluorescence intensity in the tumor and major organs at 12 h and 24 h post-injection based on the images shown in Fig. 5C. **Figure S14.** Distribution of RHE in each tissue from the RHE group and RD NPs group at 12 h (A) and 24 h (B) post-injection. Distribution of DOX in each tissue from the DOX group and RD NPs group at 12 h (C) and 24 h (D) post-injection. **Figure S15.** Average numbers of surface lung metastatic lesions in the images shown in Fig. 6E. **Figure S16.** Immunohistochemical staining for NF-κB P65, MMP-9, Bcl-2 and Bax in tumor tissues from the treated groups. **Figure S17.** Quantitative analysis of the relative intensities of the NF-κB P65, MMP-9, Bcl-2 and Bax bands in the images shown in Fig. 6F. **Table S1**. Release kinetics of RHE/DOX NPs *in vitro*.

## Data Availability

All data generated or analyzed during this study are included in this published article.

## Abbreviations

RHE: Rhein
DOX: Doxorubicin
RD NPs: RHE/DOX nanoparticles
MD: Molecular dynamics
CI: combination index
HCPT: 10-Hydroxycamptothecin
GAFF: The generalized Amber force field
RESP: The restrained electrostatic potential
RHE/DOX: RHE and DOX
DLS: Dynamic light scattering
TEM: Transmission electron microscope
SEM: Scanning electron microscope
EE: Encapsulation efficiency
PDI: Polydispersity index
FBS: Fetal bovine serum
PBS: Phosphate-buffered saline
DMEM: Dulbecco’s Modified Eagle’s Medium
CLSM: Confocal laser scanning microscopy
UPLC-MS/MS: Ultra-performance liquid chromatography-tandem mass spectrometry
TIR: Tumor inhibition rate
H&E: Hematoxylin and eosin
SD: Standard deviations
SASA: Solvent-accessible surface areas
*AUC*_*0*-*∞*_: The area under the curve
RES: Reticuloendothelial system
cTnI: Cardiac troponin I
ALT: Alanine aminotransferase
BUN: Blood urea nitrogen
CRE: Creatinine
UA: Uric acid
