# Supplementary material for: Synergistic inhibition of metastatic breast cancer by dual-chemotherapy with excipient-free rhein/DOX nanodispersions
Source: J Nanobiotechnology. 2020 Aug 26;18:116. doi: 10.1186/s12951-020-00679-2 (PMC7449082; doi:10.1186/s12951-020-00679-2)
Supplement: Supplementary file 1 — Additional file 1: Figure S1. The process of RHE and DOX co-assembly in an aqueous solution extracted from the MD simulation. Figure S2. (A) The numbers of intermolecular hydrogen bonds and (B) π-π stacking interactions between DOX and RHE molecules during the MD simulation. Figure S3. (A) The SASA of the assembled clusters and (B) the number of hydrogen bonds between the clusters and solvent water during the MD simulation. Figure S4. The assembly of clusters of pure DOX (left panel) and pure RHE (right panel) in aqueous solution after an 11-ns MD simulation. Figure S5. Numbers of π-π stacking interactions and intermolecular hydrogen bonds formed in the co-assembled clusters of DOX and RHE, and self-assembled clusters of DOX and RHE alone, respectively. Figure S6. The size of RD NPs. Figure S7. The zeta potential of RD NPs. Figure S8. The particle size and PDI of RD NPs during storage at room temperature. Figure S9. In vitro cytotoxicity of 4T1 cells treated with (A) RHE, (B) DOX, (C) RHE/DOX and RD NPs for 48 h. Figure S10. Quantitative analysis of the scratch healing rate based on the images shown in Fig. 4A. Figure S11. Quantitative analysis of migrating or invading cells based on the images shown in Fig. 4B and 4C. Figure S12. Quantitative analysis of the relative intensities of the NF-κB P65, MMP-9, Bcl-2 and Bax bands based on the images shown in Fig. 4E. Figure S13. Semiquantitative analysis of the ex vivo DOX fluorescence intensity in the tumor and major organs at 12 h and 24 h post-injection based on the images shown in Fig. 5C. Figure S14. Distribution of RHE in each tissue from the RHE group and RD NPs group at 12 h (A) and 24 h (B) post-injection. Distribution of DOX in each tissue from the DOX group and RD NPs group at 12 h (C) and 24 h (D) post-injection. Figure S15. Average numbers of surface lung metastatic lesions in the images shown in Fig. 6E. Figure S16. Immunohistochemical staining for NF-κB P65, MMP-9, Bcl-2 and Bax in tumor tissues from the tr [file 12951_2020_679_MOESM1_ESM.docx]

**Supporting information**

Synergistic inhibition of metastatic breast cancer by dual-chemotherapy with excipient-free rhein/DOX nanodispersions

*Ruoning Wang,*^1,2,†^ *Yujie Yang,* ^1,2,†^ *Mengmeng Yang,* ^1,2^ *Dandan Yuan,*^1^

*Jinyu Huang,*^1,2^ *Rui Chen,*^1^ *Honglan Wang,*^1,2^ *Lihong Hu,*^1,3^ *Liuqing Di,*^1,2^ *Junsong Li,*^1,2,*^

1 School of Pharmacy, Nanjing University of Chinese Medicine, Nanjing, China
2 Jiangsu Engineering Research Center for Efficient Delivery System of TCM, Nanjing, China
3 Jiangsu Key Laboratory for Functional Substance of Chinese Medicine, Nanjing, China

† Contributed equally to this work.

Corresponding authors: Junsong Li

E-mail: lijunsong1964@163.com

Postal address: School of Pharmacy, Nanjing University of Chinese Medicine, 138 Xianlin Avenue, Nanjing 210023, China


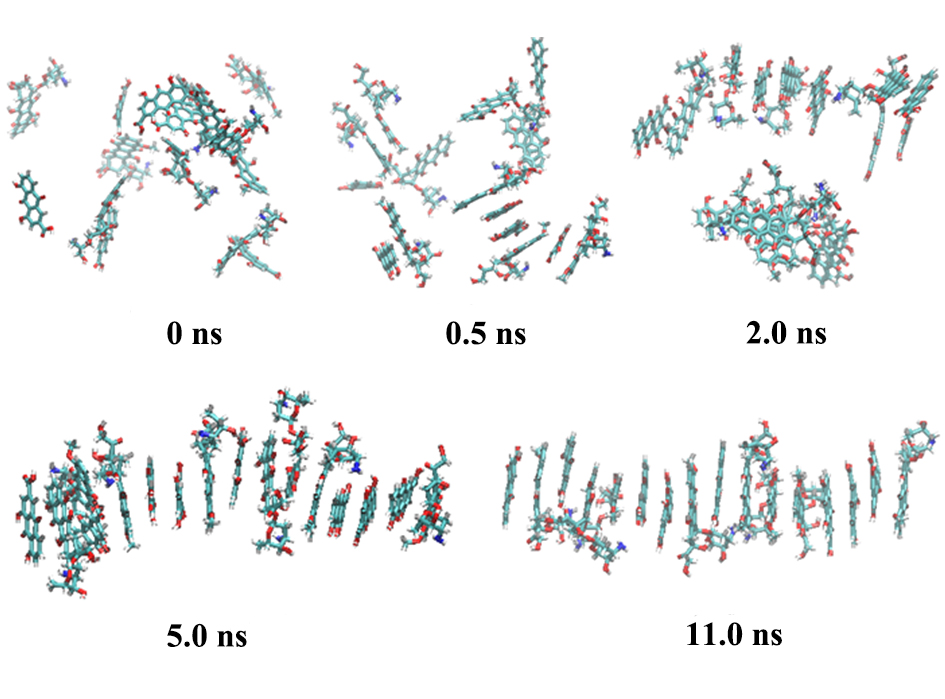


**Figure S1.** The process of RHE and DOX co-assembly in an aqueous solution extracted from the MD simulation. The solvent water molecules are omitted for simplicity.


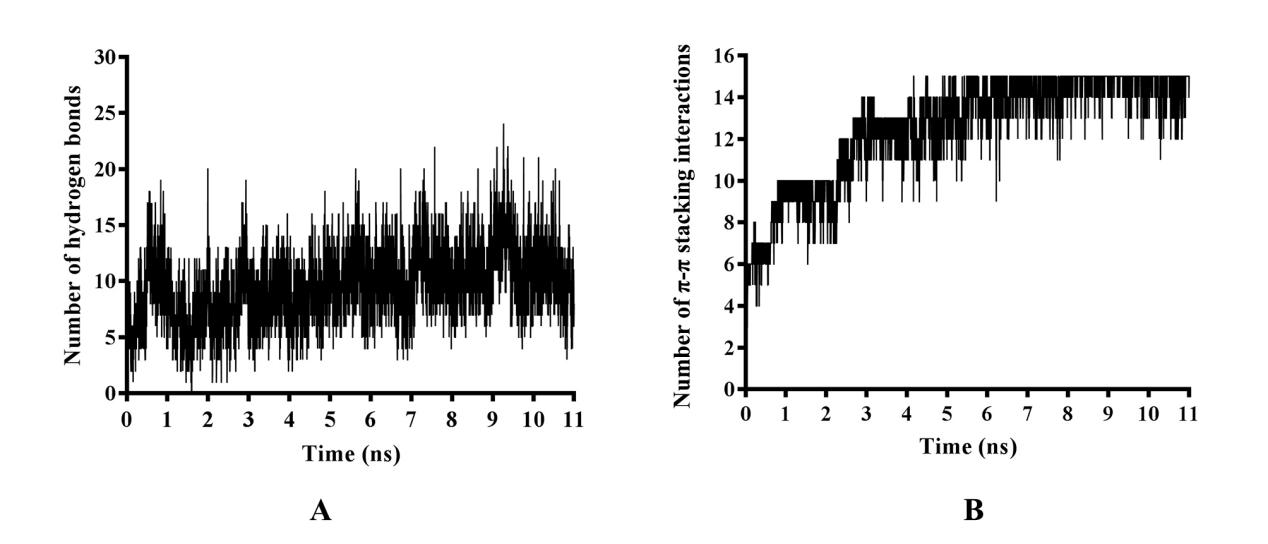


**Figure S2.** (A) The numbers of intermolecular hydrogen bonds and (B) π-π stacking interactions between DOX and RHE molecules during the MD simulation. A hydrogen bond is defined if the distance between the donor and acceptor is less than 4.0 Å and the corresponding angle is larger than 120°. The criterion for a π-π stacking interaction is that the distance between two aromatic rings is less than 5.0 Å.


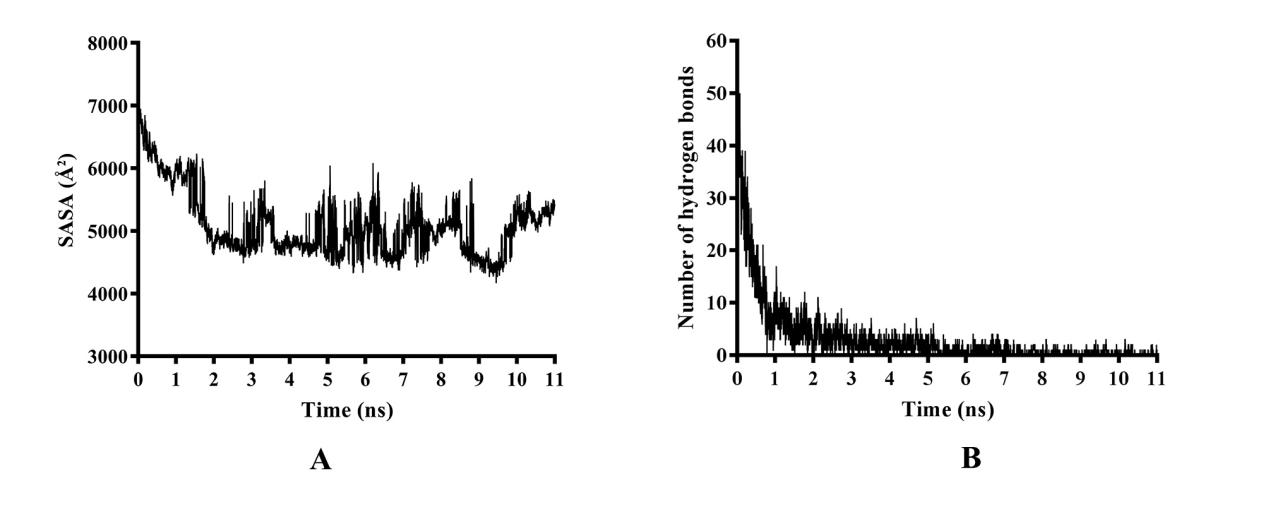


**Figure S3.** (A) The SASA of the assembled clusters and (B) the number of hydrogen bonds between the clusters and solvent water during the MD simulation.


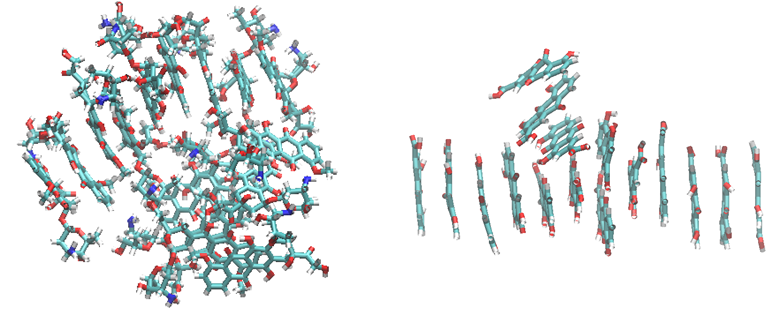


**Figure S4.** The assembly of clusters of pure DOX (left panel) and pure RHE (right panel) in aqueous solution after an 11 ns MD simulation. The solvent water molecules are omitted for simplicity.


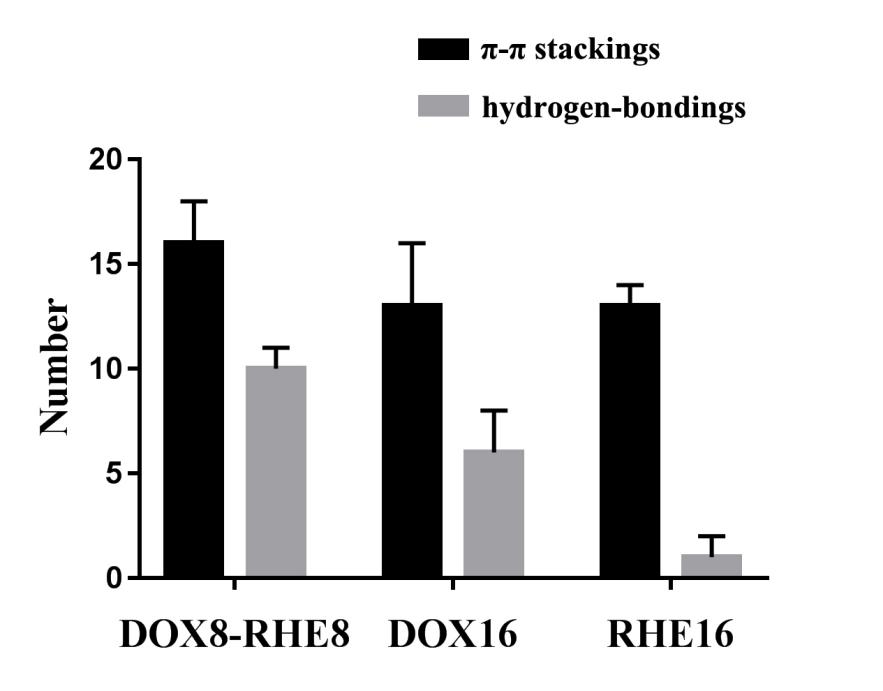


**Figure S5.** Numbers of π-π stacking interactions and intermolecular hydrogen bonds formed in the co-assembled clusters of DOX and RHE, and self-assembled clusters of DOX and RHE alone, respectively, after the 11 ns MD simulation. 8 DOX and 8 RHE molecules, and 16 DOX molecules and 16 RHE molecules immersed in 2000 water molecules were analyzed in the MD simulations of the RD NPs, pure DOX and pure RHE, respectively.


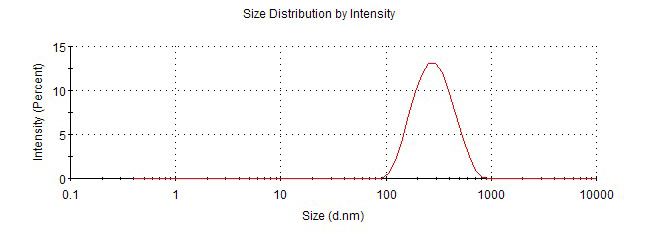


**Figure S6.** The size of RD NPs.


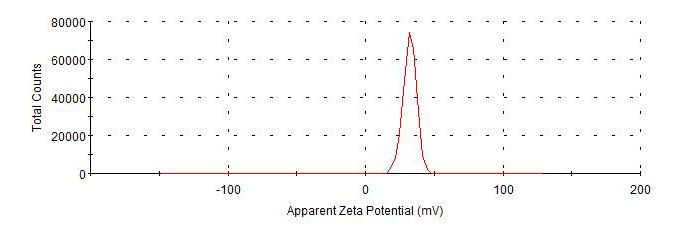


**Figure S7.** The zeta potential of RD NPs.


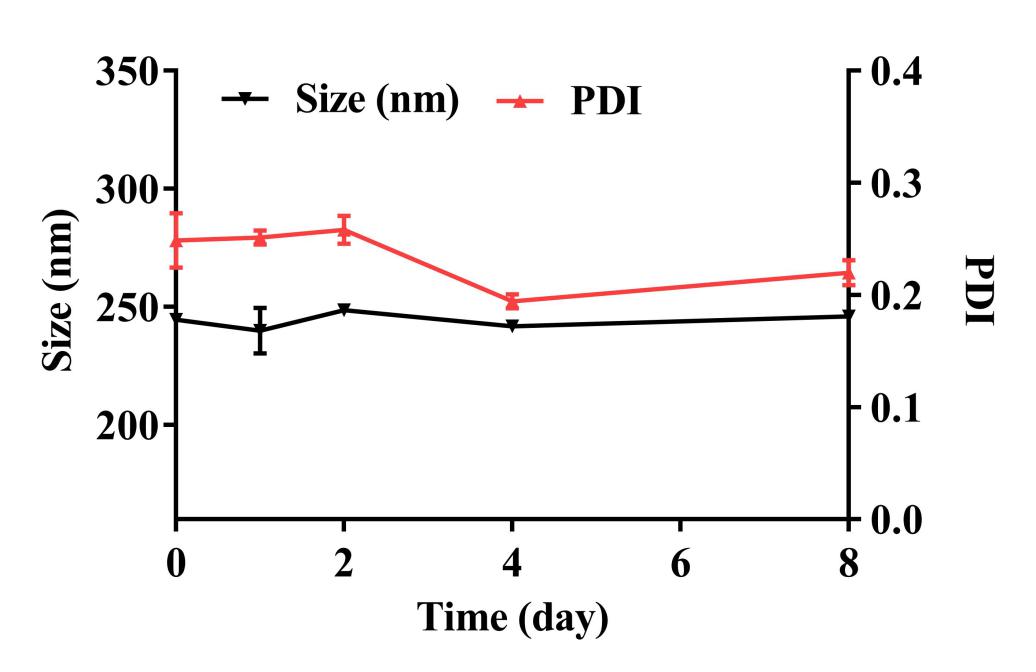


**Figure S8.** The particle size and PDI of RD NPs during storage at room temperature.

**Table S1.** Release kinetics of RHE/DOX nanoparticles *in vitro*.

| Drugs | Buffer solutions | Zero-order | | First-order | | Higuchi | |
| --- | --- | --- | --- | --- | --- | --- | --- |
|  |  | regression equation | R^2^ | regression equation | R^2^ | regression equation | R^2^ |
| RHE from RD NPs | pH 7.4 | M_t_=3.72t+18.43 | 0.76 | M_t_=89.09×（1-e^-0.17t^） | 0.99 | M_t_=20.41t^1/2^+0.64 | 0.95 |
|  | pH 5.0 | M_t_=3.16t+9.21 | 0.88 | M_t_=80.22×（1-e^-0.10t^） | 0.99 | Mt=16.48t^1/2^-4.25 | 0.98 |
| DOX from RD NPs | pH 7.4 | M_t_=3.67t+13.87 | 0.80 | M_t_=88.04×（1-e^-0.14t^） | 0.99 | M_t_=19.71t^1/2^-2.87 | 0.96 |
|  | pH 5.0 | M_t_=3.94t+23.452 | 0.64 | M_t_=93.48×（1-e^-0.24t^） | 0.99 | M_t_=22.45t^1/2^+2.95 | 0.89 |


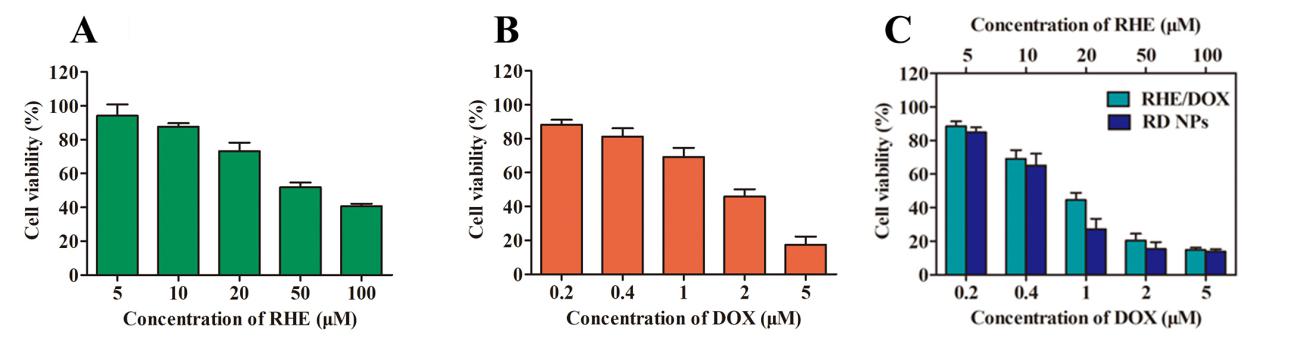


**Figure S9.** *In vitro* cytotoxicity of 4T1 cells treated with (A) RHE, (B) DOX, (C) RHE/DOX and RD NPs for 48 h.


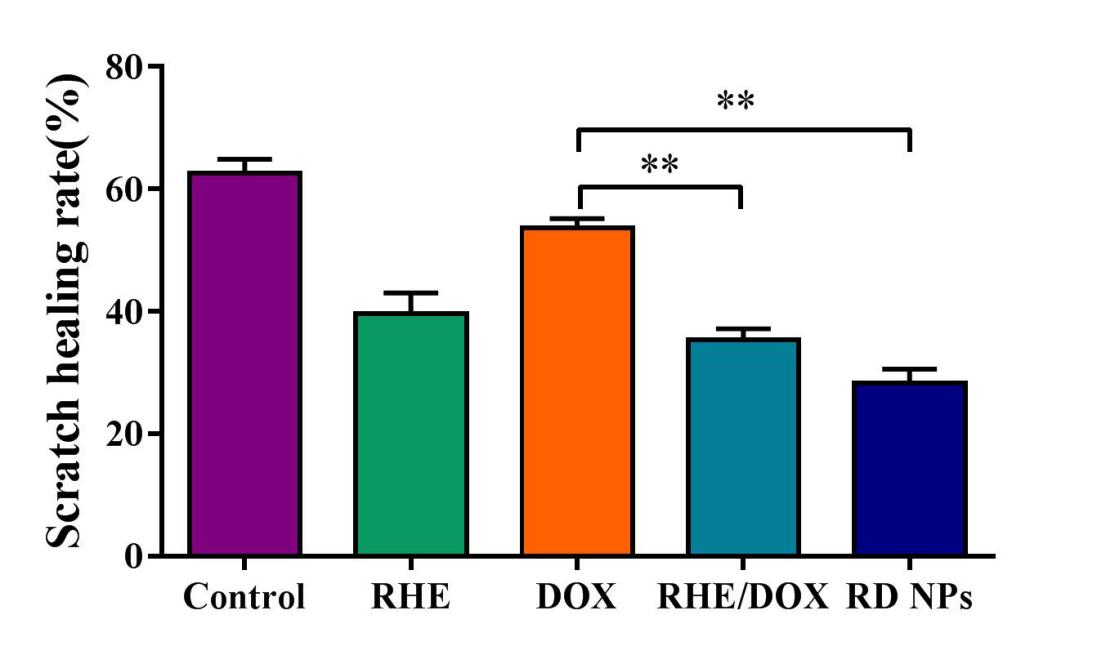


**Figure S10.** Quantitative analysis of the scratch healing rate based on images in Fig. 4A.


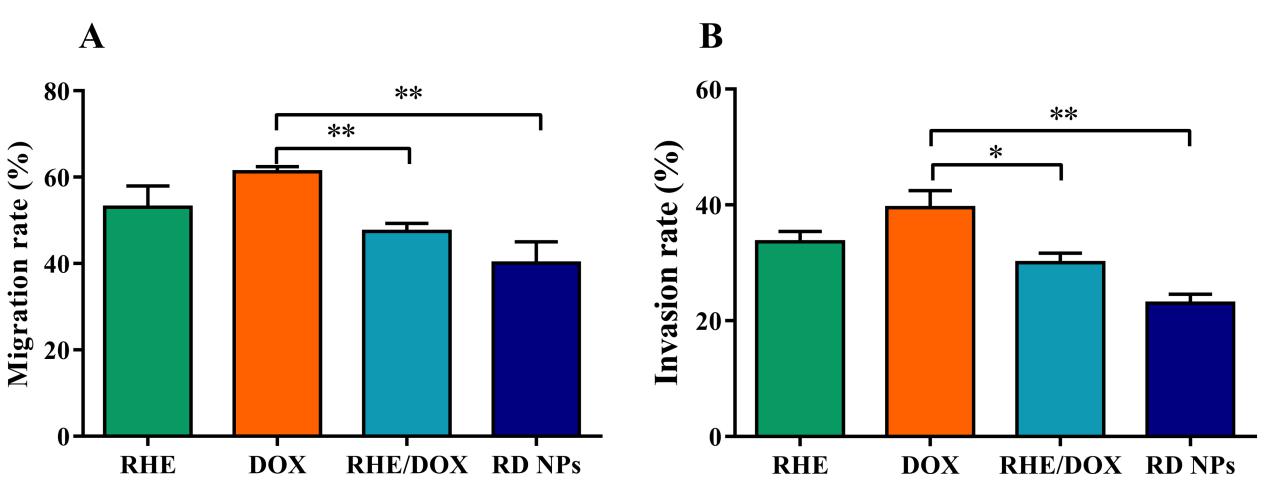


**Figure S11.** Quantitative analysis of migrating (A) or invading cells (B) based on images in Fig. 4B and 4C.


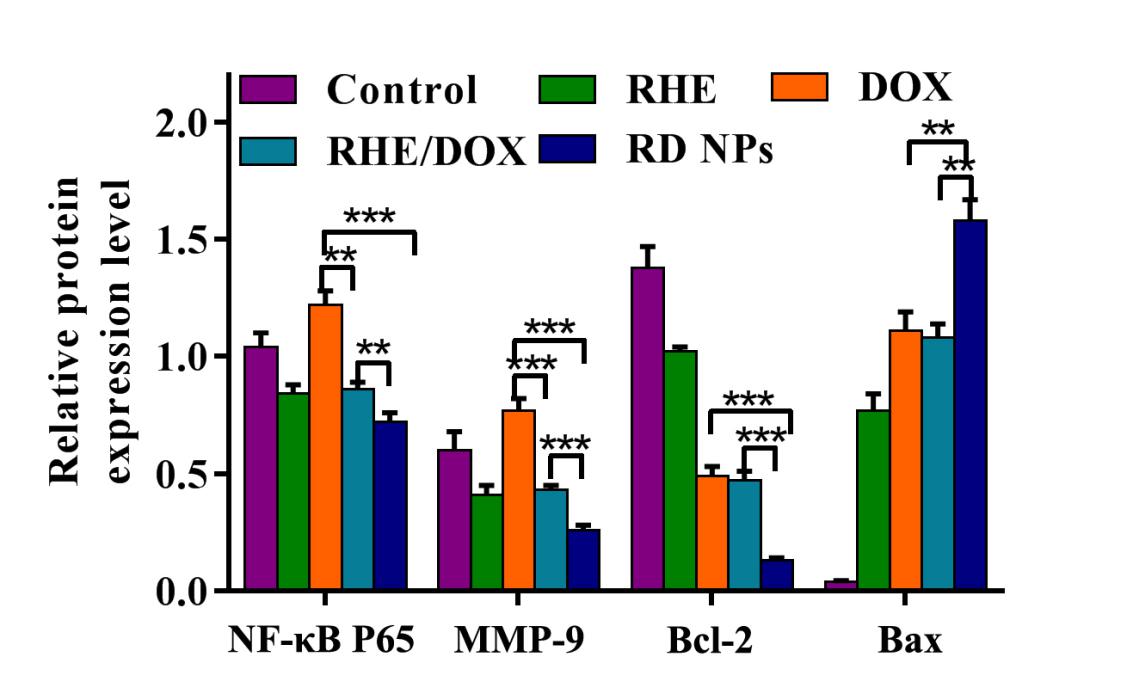


**Figure S12.** Quantitative analysis of the relative intensities of NF-κB P65, MMP-9, Bcl-2 and Bax based on images in Fig. 4E. (*n* = 3, mean ± SD). ***p* < 0.01, ****p* < 0.001.


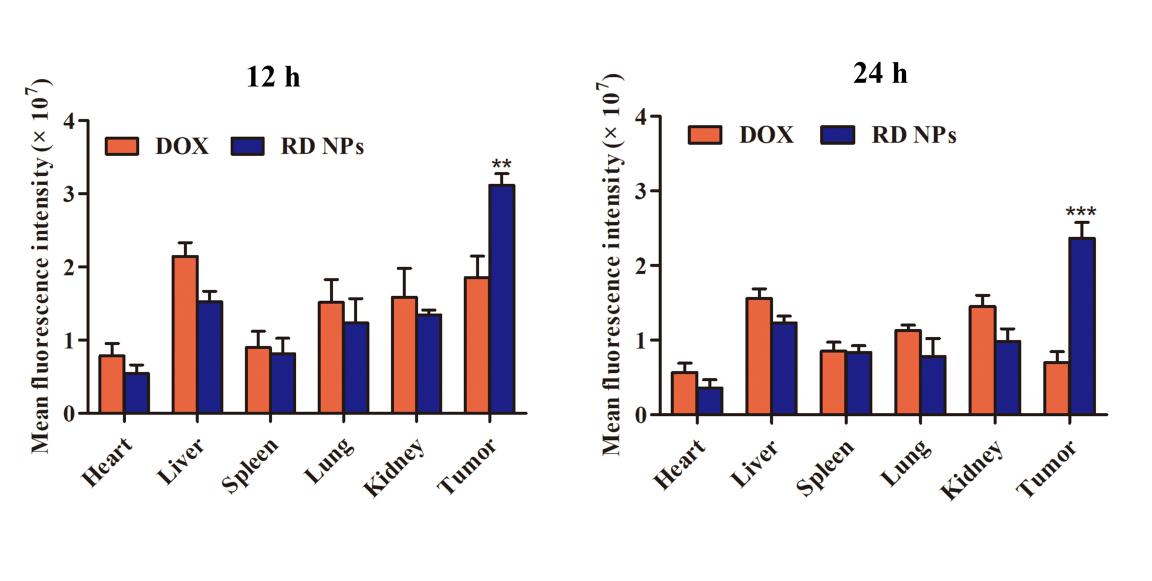


**Figure S13.** Semiquantitative analysis of the *ex vivo* DOX fluorescence intensity in the tumor and major organs at 12 h and 24 h post-injection based on images in Fig. 5C. (n = 3, mean ± SD). ***p* < 0.01, ****p* < 0.001.


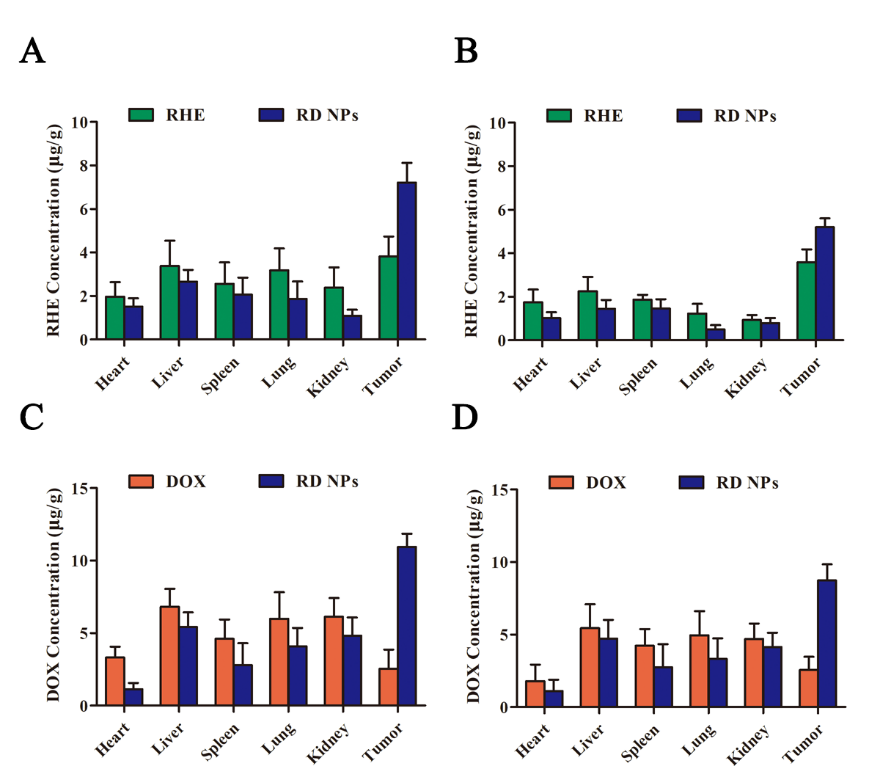


**Figure S14.** Distribution of RHE in each tissue from the RHE group and RD NPs group in each tissue at 12 h (A) and 24 h (B) post-injection. Distribution of DOX in each tissue from the DOX group and RD NPs group in each tissue at 12 h (C) and 24 h (D) post-injection.


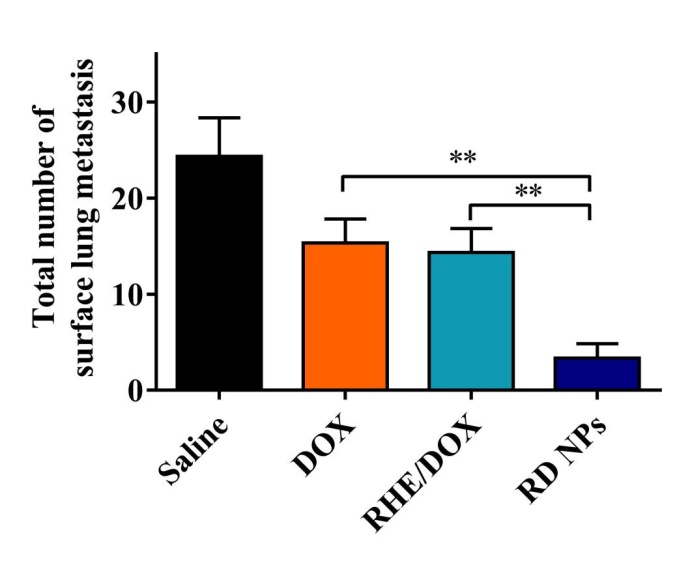


**Figure S15.** Average numbers of surface lung metastatic lesions in the images shown in Fig. 6E (n=5, mean ± SD).


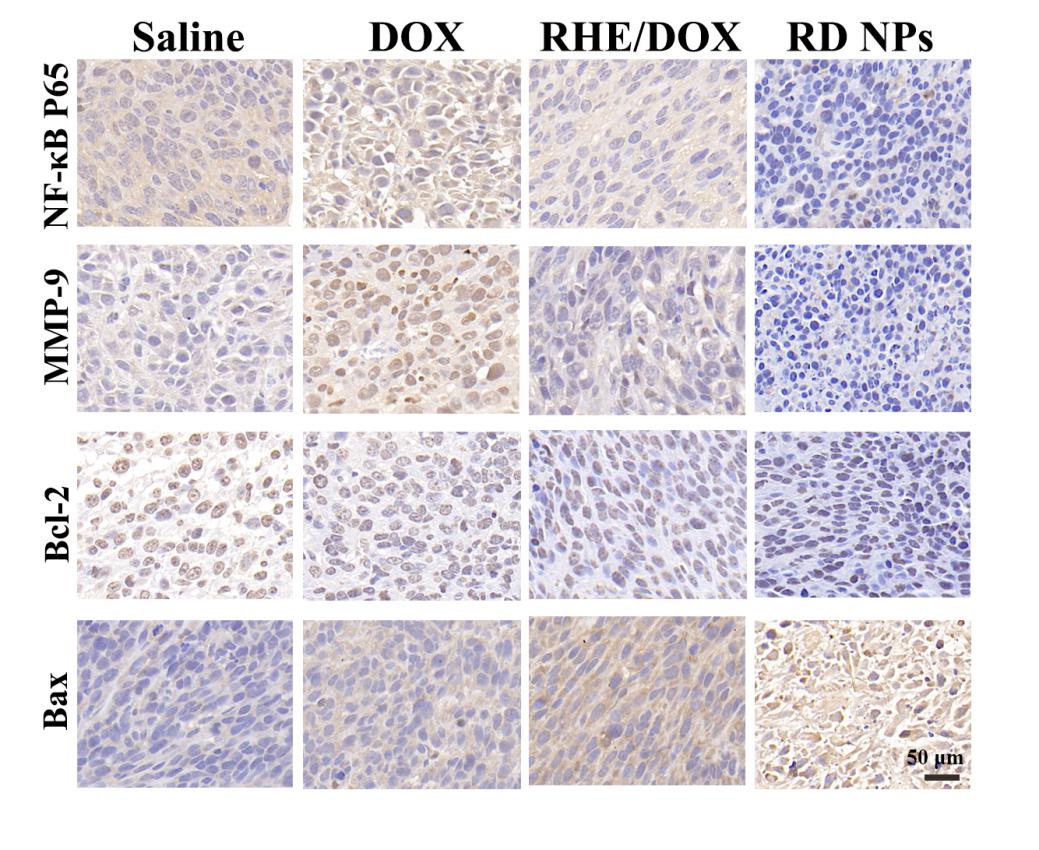


**Figure S16.** Immunohistochemical staining for NF-κB P65, MMP-9, Bcl-2 and Bax in tumor tissues from the treated groups.


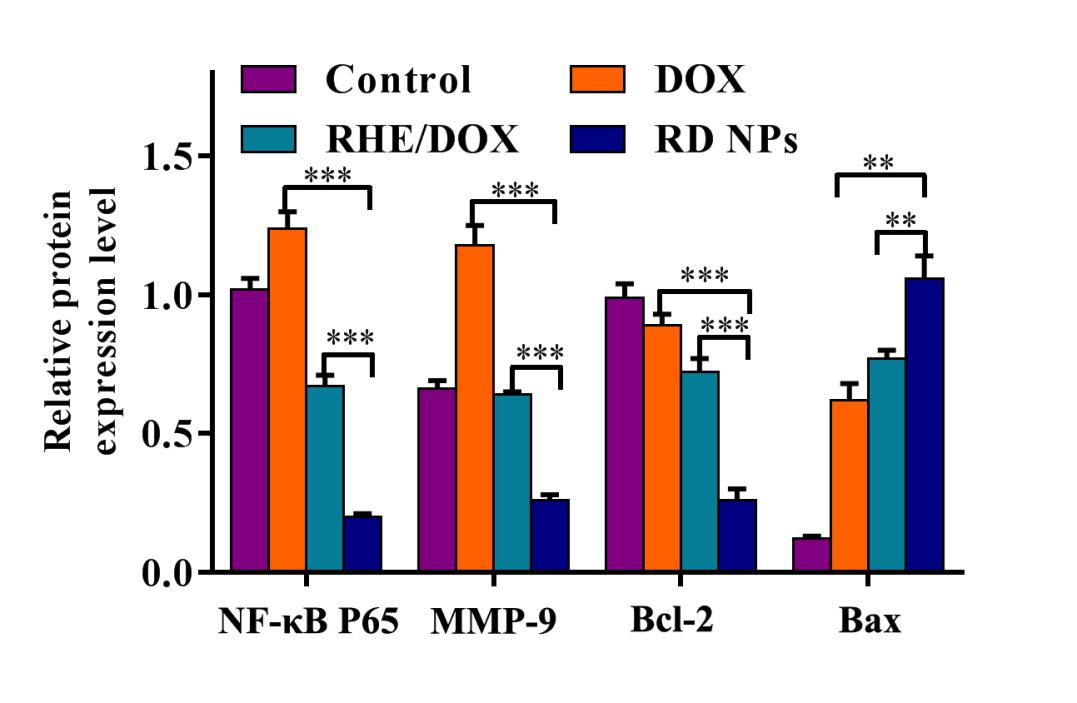


**Figure S17.** Quantitative analysis of the relative intensities of the NF-κB P65, MMP-9, Bcl-2 and Bax bands in the images shown in Fig. 6F (n = 3, means ± SD). **p <* 0.05, ***p <* 0.01, and ****p <* 0.001
